# Real-world performance of open-source large language models in diabetes diagnosis

**DOI:** 10.3389/fendo.2026.1747468

**Published:** 2026-03-25

**Authors:** Shuting Yang, Sujie Liu, Yuxi Ma, Baowen Gai, Junwei Liu, Liansheng Wang, Feng Gao, Zhiguang Zhou

**Affiliations:** 1 National Clinical Research Center for Metabolic Diseases, Key Laboratory of Diabetes Immunology (Central South University), Ministry of Education, and Department of Metabolism and Endocrinology, The Second Xiangya Hospital of Central South University, Changsha, Hunan, China; 2 Department of Neurosurgery, The Sixth Affiliated Hospital of Sun Yat-sen University, Guangzhou, China; 3 Graceland Medical Center, The Sixth Affiliated Hospital, Sun Yat-sen University, Guangzhou, Guangdong, China; 4 Department of Computer Science at the School of Informatics, Xiamen University, Xiamen, China; 5 National Institute for Data Science in Health and Medicine, Xiamen University, Xiamen, China; 6 Department of Colorectal Surgery, Department of General Surgery, The Sixth Affiliated Hospital, Sun Yat-sen University, Guangzhou, Guangdong, China; 7 Guangdong Provincial Key Laboratory of Colorectal and Pelvic Floor Diseases, The Sixth Affiliated Hospital, Sun Yat-sen University, Guangzhou, Guangdong, China; 8 Biomedical Innovation Center, The Sixth Affiliated Hospital, Sun Yat-sen University, Guangzhou, Guangdong, China; 9 Guangzhou National Laboratory, Guangzhou, China

**Keywords:** artificial intelligence, diabetes, diabetes complication disease, diagnosis, large language model

## Abstract

**Background:**

This study aimed to evaluate the performance of diverse open-source large language models (LLMs) in diagnosing diabetes subtypes and comorbidities from unstructured clinical text, assessing the impact of model characteristics, prompting, and language.

**Methods:**

We conducted a retrospective analysis of 11,329 adult diabetes patients from a large Chinese tertiary center (2010–2020). Various open-source LLMs were tested using four prompting strategies in English and Chinese. Primary outcomes were F1-scores for multi-class diabetes subtyping and binary classification of diabetic kidney disease (DKD) and metabolic syndrome (MetS).

**Results:**

LLMs demonstrated high performance in complex subtyping (peak F1 0.951) but showed limitations in rule-based DKD (F1 0.570) and MetS (F1 0.650) diagnosis. Chain-of-Thought prompting improved MetS classification but degraded DKD performance. Optimal model size was approximately 32B parameters. Notably, English prompts outperformed Chinese prompts on native Chinese text.

**Conclusion:**

Open-source LLMs exhibit strong holistic pattern recognition for complex classification but struggle with rule-based procedural reasoning. These models are promising as clinical co-pilots to augment expert decision-making rather than serving as autonomous diagnostic tools.

## Introduction

Effective management of Diabetes Mellitus (DM) hinges on the accurate diagnosis of its subtypes and comorbidities, a process often challenged by overlapping phenotypes and complex criteria that can lead to poor patient outcomes (1, 2).

Large Language Models (LLMs) are transforming medical informatics (3). While initial research on proprietary models confirmed their potential (4, 5), the recent proliferation of open-source LLMs offers greater transparency and customizability, making them a highly promising approach for specialized medical tasks (6). This has spurred the creation of comprehensive specialized medical benchmarks, such as MedQA and PubMedQA, which show that leading open-source models can be competitive, especially with effective prompting (7–9). However, while these standardized datasets have advanced the field, a critical gap remains: the evaluation of LLMs on real-world, non-English clinical narratives is still underexplored. The complex reasoning required for such high-stakes tasks using real-world data is therefore underexplored.

To address the critical gap, the primary objective of this study is to evaluate and compare the diagnostic performance of various open-source LLMs across three specific endocrinological tasks (diabetes subtyping, diabetic kidney disease, and metabolic syndrome) using a large dataset of 11,329 real-world Chinese clinical narratives. Furthermore, our secondary objectives are to systematically analyze the impact of advanced prompting strategies like Chain-of-Thought (CoT) (10), model characteristics (size, family, and fine-tuning), and language-specific tuning on diagnostic accuracy.

## Materials and methods

### Study population

This retrospective, cross-sectional study was approved by the Ethics Committee of the Second Xiangya Hospital, Central South University. We analyzed de-identified discharge summaries from inpatients in the endocrinology department between January 2010 and December 2020. Inclusion was based on a specialist-confirmed diabetes diagnosis and complete records; patients with other confounding primary diseases were excluded. Patient confidentiality was maintained through a standardized de-identification process. The study was approved by the institutional ethics committee and adhered to the Declaration of Helsinki.

### Disease definitions

We defined all diagnostic outcomes based on established clinical guidelines. Diabetes mellitus (DM) classification was determined through a comprehensive clinical assessment that included onset characteristics, disease progression, autoantibody status, islet function, genetic testing, and treatment response, ultimately categorizing patients into type 1 DM (T1DM), type 2 DM (T2DM), specific types of diabetes (SD), or gestational DM (GDM), in alignment with American Diabetes Association (ADA) principles (11). Diabetic kidney disease (DKD) was diagnosed according to ADA criteria, defined as persistent albuminuria (urinary albumin-to-creatinine ratio [UACR] ≥30 mg/g or 24-hour albumin excretion rate [AER] ≥20 μg/min) for over three months, after the exclusion of other nephropathies (12). Metabolic syndrome (MetS) was identified using the 2017 Chinese Diabetes Society (CDS) criteria, requiring the presence of at least three of the following five components: 1) central obesity (waist circumference ≥90 cm for men, ≥85 cm for women); 2) hyperglycemia (fasting plasma glucose [FPG] ≥6.1 mmol/L, 2-hour post-load glucose ≥7.8 mmol/L, or diagnosed diabetes); 3) hypertension (blood pressure ≥130/85 mmHg or receiving antihypertensive therapy); 4) hypertriglyceridemia (fasting triglycerides [TG] ≥1.70 mmol/L); and 5) low high-density lipoprotein cholesterol (HDL-C) (<1.04 mmol/L) (13).

### LLM selection and setting

We selected a diverse set of open-source LLMs based on four key principles. The selection covered: 1) a wide range of parameter scales (2B to 72B); 2) diverse architectures [Llama (14), Qwen (15–18), DeepSeek (19, 20)]; 3) a balance between general-purpose models and medically fine-tuned counterparts [MMedLM2 (21), HuatuoGPT-2 (22)]; and 4) models with strong community support and available quantized versions to ensure practical relevance (**Supplementary Appendix 1**). Model deployment was scaled-dependent. Larger models including Qwen2.5-72B, DeepSeek-32B were accessed via APIs, while smaller models were run locally on A100/RTX 3090 GPUs using the Hugging Face Transformers library, accelerated by vLLM and int4/fp8 quantization. Key inference parameters were set (temperature: 0.35–0.6, top_p: 0.7, max_tokens: 64–2048), with unspecified parameters at their defaults (**Supplementary data sheet 2**).

### Data preparation

**Figure 1:**
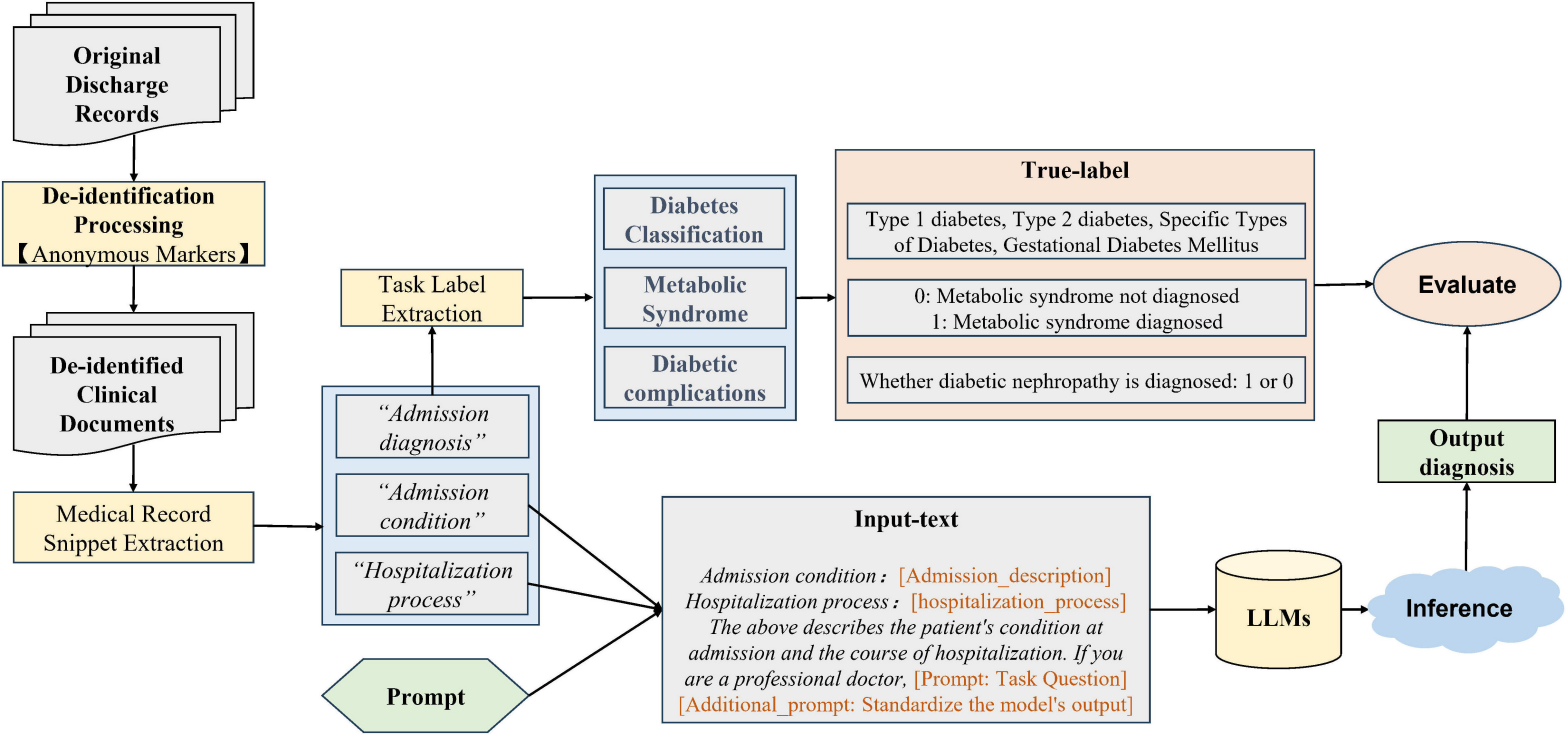
Overview of the LLM-based pipeline for clinical diagnosis. Original patient records were first anonymized and processed to extract relevant text. This text was then used to construct a task-specific prompt that queries a LLM. The LLM performed inference to produce a diagnosis, which was then evaluated for accuracy against true labels.

We developed a systematic pipeline to transform raw clinical text from diabetes discharge summaries into a structured format for LLM evaluation. Initially, regular expressions were used to extract the distinct sections including “Admission Diagnosis,” “Admission Condition,” and “Hospitalization Process” sections from each record. Next, ground-truth labels for three tasks (diabetes classification, metabolic syndrome, and diabetic kidney disease) were automatically generated from the “Admission Diagnosis” text via pattern matching and predefined keyword dictionaries. The extracted narratives and labels were then integrated into a tabular dataset, which was subsequently deduplicated based on a unique health event ID. Crucially, to rigorously prevent label leakage, a prevalent issue where explicit diagnostic terms in the input artificially inflate model performance, the “Admission Diagnosis” field was strictly masked and excluded from the downstream LLM prompts (23). Finally, for model input, only the unstructured narrative sections of the “Admission Condition” and “Hospitalization Process” were concatenated and appended with a standardized, task-specific prompt, forcing the models to deduce diagnoses entirely from contextual clinical descriptions (**Figure 1**).To evaluate the models’ performance under true real-world clinical conditions, missing critical variables were not explicitly masked or annotated with placeholders in the input text. Instead, the LLMs were provided with the raw, unstructured clinical narratives. Consequently, the models were required to independently process the text and infer the absence of these variables if they were not explicitly documented.

We used a three-tiered protocol to standardize model predictions for evaluation. Tier 1 used prompt engineering to constrain outputs to a predefined format. For non-conforming outputs, Tier 2 applied a rule-based parser with keyword matching. Any remaining ambiguous outputs were adjudicated in Tier 3. Critically, to preserve clinical nuance, detailed analytical responses from medical-specific LLMs were exempted from this forced categorization and instead retained for qualitative analysis, ensuring both robust quantitative metrics and valuable qualitative insights (**Figure 1**).

### Prompting strategies

We evaluated four prompting strategies of progressively increasing complexity to assess their impact on model performance. These strategies ranged from a baseline Zero-shot direct query (Prompt 1) to Reference-guided prompting (Prompt 2) that provided a specific guide, manual, or standard framework. We further incorporated Criteria-driven prompting (Prompt 3), and finally employed Chain-of-Thought (CoT) prompting (Prompt 4) to elicit a step-by-step rationale prior to the final diagnosis. This hierarchical framework enabled a systematic analysis of the model’s response to varying levels of guidance (**Table 1**) (**Supplementary data sheet 3**).

**Table 1:** Summary of prompting strategies.

Prompt	Strategy name	Description	Key feature
Prompt 1	Zero-Shot Direct Query	A simple, direct question posed to the model	Minimal guidance
Prompt 2	Reference-Guided Prompting	Providing a specific guide, manual, or standard framework.	Anchoring the output to an authoritative standard
Prompt 3	Criteria-Driven Prompting	Providing explicit instructions and a specific list of criteria.	Specifying evaluation rubrics
Prompt 4	Chain-of-Thought (CoT) Prompting	Guides the model to provide a step-by-step analysis before the final answer	Elicits explicit reasoning process

### Statistical analysis

Model performance was assessed using standard classification metrics (accuracy, precision, recall, F1-score). We prioritized the macro-F1 score for the multi-class task and the F1-score for binary tasks. Statistical robustness was ensured by calculating 95% confidence intervals (CIs) via bootstrapping (n=1,000). To evaluate the statistical significance of pairwise performance differences, McNemar’s test (p<0.05) was applied. Specifically, for Task 1, it was utilized to compare the efficacy of different prompting strategies on the top-performing model, as well as to assess performance variations among distinct LLMs under an optimal prompt. Additionally, a sensitivity analysis on balanced data subsets was conducted to evaluate performance on rare classes. All analyses utilized Python’s scikit-learn and statsmodels libraries.

## Results

### Characteristics of study populations

A total of 11,329 patients with diabetes were initially included. After excluding 1,488 patients with unclassified subtypes, 9,841 patients were included in the final diabetes subtyping analysis. The distribution was as follows: 8,911 (90.5%) with T2DM, 737 (7.5%) with T1DM, 147 (1.5%) with specific types of diabetes (SD), and 46 (0.5%) with GDM. Separately, the entire initial cohort (n = 11,329) was assessed for comorbidities, revealing 3,753 cases of metabolic syndrome (MetS) and 2,374 cases of diabetic kidney disease (DKD).

### Overall performance

We comprehensively evaluated the performance of a series of open-source LLMs on three core diabetes-related diagnostic tasks. In the diabetes classification task (Task 1), the DeepSeek-V3 model with Chinese Prompt 1 achieved the best F1 score of 0.951. For the DKD diagnosis task (Task 2), the DeepSeek-R1 model combined with Chinese Prompt 2 performed best, achieving an F1 score of 0.570. In the metabolic syndrome diagnosis task (Task 3), the DeepSeek-32B model paired with Chinese Prompt 3 obtained the highest F1 score of 0.650. The initial findings revealed the optimal performance of DeepSeek models.

### Performance by task

#### Task 1: diabetes classification diagnosis

**Figure 2:**
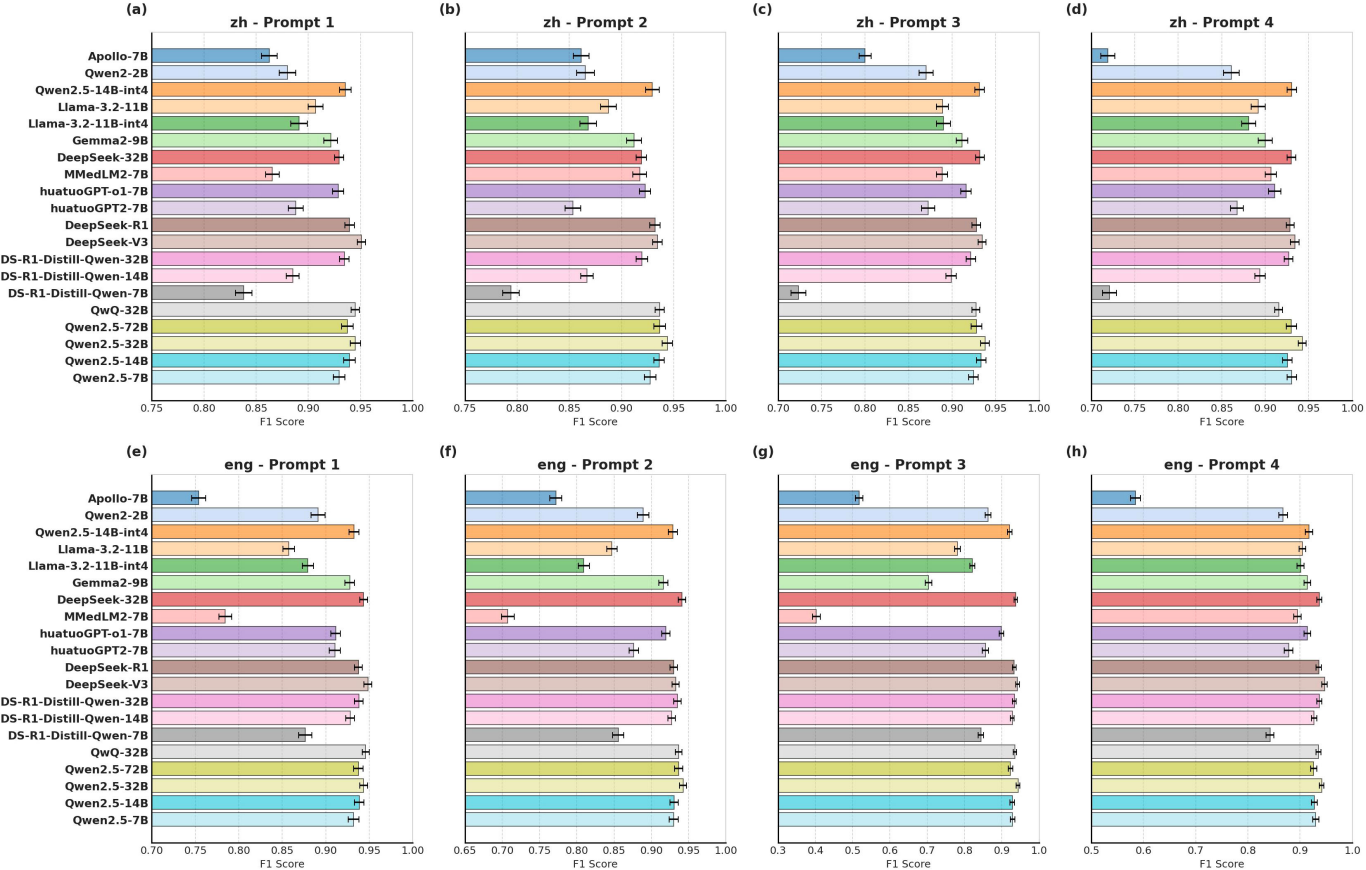
Distribution of weighted F1-scores (95%CIs) for different models on the diabetes subtyping task. This figure showed the distribution of Weighted F1-scores (95%CIs) for different models on the diabetes subtyping task using **(a)** Chinese prompt 1, **(b)** English prompt 1, **(c)** Chinese prompt 2, **(d)** English prompt 2, **(e)** Chinese prompt 3, **(f)** English prompt 3, **(g)** Chinese prompt 4, and **(h)** English prompt 4.

For the primary task of diabetes classification, we evaluated all models using accuracy, macro F1-score, and weighted F1-score. The DeepSeek-V3 model, when paired with Chinese Prompt 1, achieved the highest overall accuracy (95.21%) and weighted F1-score (0.951; 95%CI [0.947-0.955]) (**Supplementary Table 1** and **Supplementary Table 2** in the **Supplementary Tables**). Across all prompt configurations, DeepSeek-V3 demonstrated exceptional performance stability, consistently maintaining high F1-scores (**Figure 2**).

**Figure 3:**
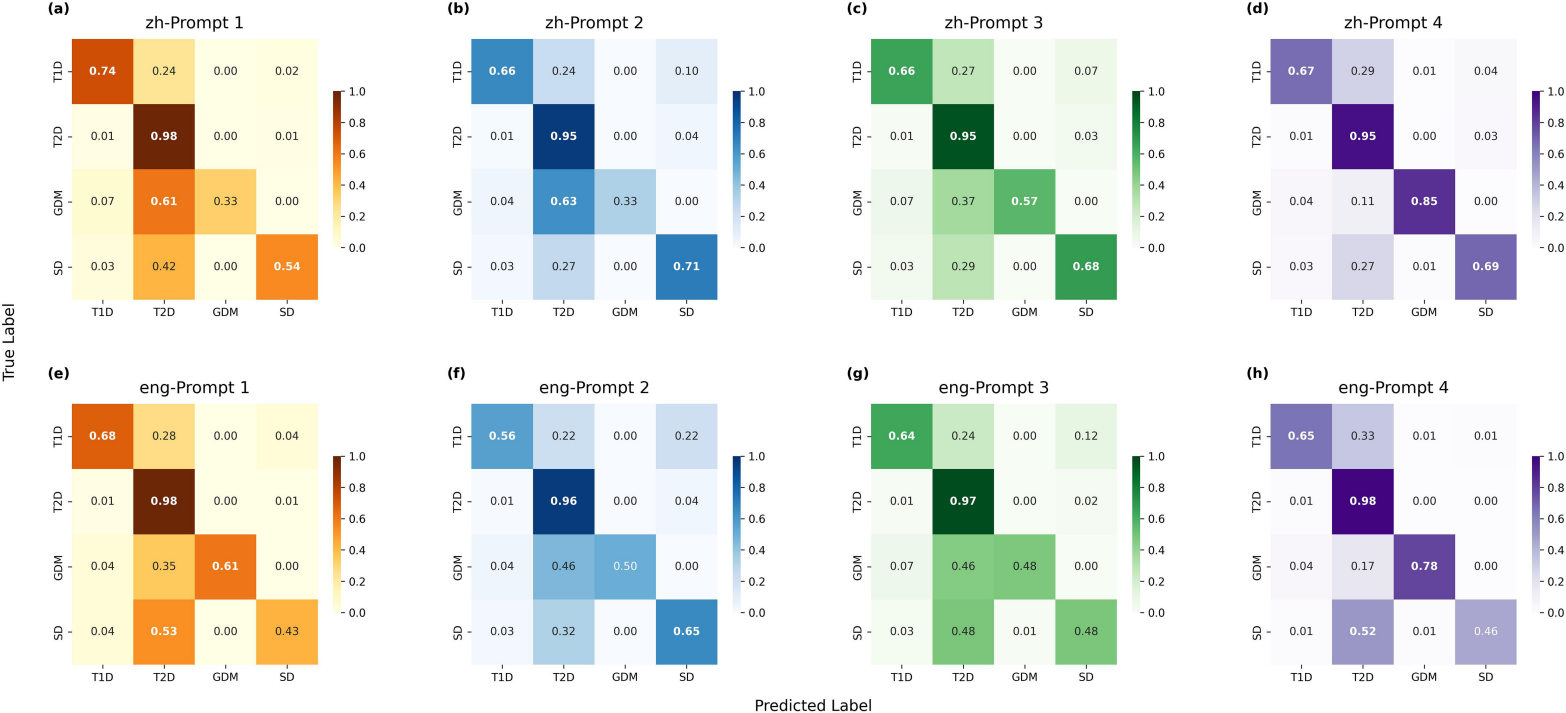
Confusion matrix for the diabetes classification task using the DeepSeek-V3 model. This figure demonstrated the confusion matrix for the diabetes classification task using the DeepSeek-V3 model with **(a)** Chinese Prompt 1, **(b)** Chinese Prompt 2, **(c)** Chinese Prompt 3, **(d)** Chinese Prompt 4, **(e)** English Prompt 1, **(f)** English Prompt 2, **(g)** English Prompt 3, **(h)** English Prompt 4.

A confusion matrix analysis revealed the top-performing model (DeepSeek-V3, Chinese Prompt 1) suffered from significant class imbalance, misclassifying high proportions of minority classes like GDM (61%) and SD (42%) as T2DM. Systematically refining the prompts proved effective. The optimal performance balance (highest macro F1-score) was achieved with DeepSeek-V3 using English Prompt 4, which markedly improved recall for minority classes and delivered the most balanced diagnostic performance, despite some residual misclassification (**Figure 3**) (**Supplementary Table 1** in the **Supplementary Tables**).

To assess robustness, a sensitivity analysis was conducted on a class-balanced subset (n=46 per class). The DeepSeek models (V3 and R1) emerged as the most robust, achieving the highest average F1-scores (69.74% and 69.67%), while models like Llama-3.2-11B showed significant performance decline (**Supplementary Figure 3** and **Supplementary Figure 4** in the **Supplementary Figures**). We also identified the performance ceiling: DeepSeek-V3 with Chinese Prompt 4 achieved the peak F1-score of 77.39%, outperforming the next-best models (**Supplementary Figure 5** in the **Supplementary Figures**). Furthermore, the analysis revealed a clear language bias, with English prompts yielding a superior mean F1-score (61.38%) compared to Chinese prompts (56.65%) across all models (**Supplementary Figure 6** and **Supplementary Figure 7** in the **Supplementary Figures**).

#### Task 2: diabetic kidney disease diagnosis

**Figure 4:**
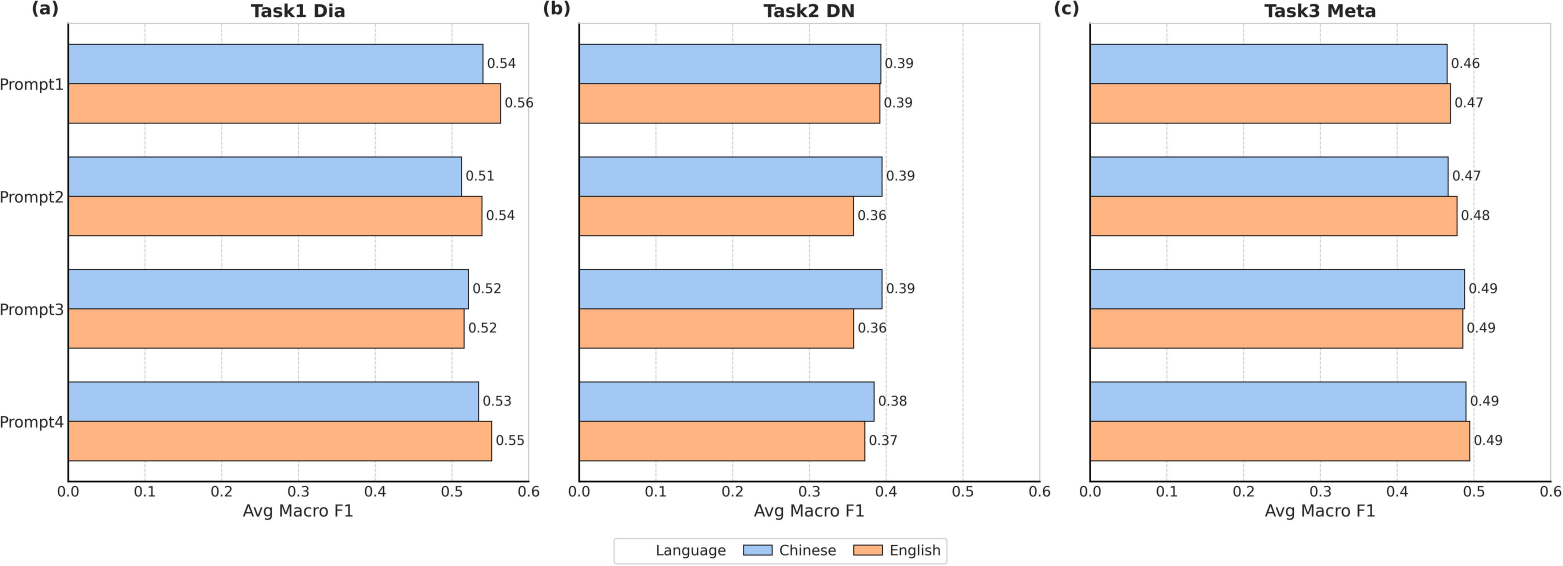
Average macro F1 comparison across tasks and prompts. **(a)** Performance of different prompts, as measured by Macro F1-score, on the diabetes classification diagnosis task (Task 1), evaluated under both Chinese and English language conditions. Prompt 1 showed the best performance in both conditions. **(b)** Performance of different prompts, as measured by Macro F1-score, on the diabetic kidney disease diagnosis task (Task 2), evaluated under both Chinese and English language conditions. **(c)** Performance of different prompts, as measured by Macro F1-score, on the metabolic syndrome diagnosis task (Task 3), evaluated under both Chinese and English language conditions.

In the DKD diagnosis task, the DeepSeek-R1 model performed best when combined with Chinese Prompt 2, achieving the highest F1 score of 0.570 (95%CI [0.556, 0.583]). And the combination of Qwen2-2B and Chinese Prompt 2 exhibited the greatest stability, as evidenced by yielding the tightest 95%CI of [0.354, 0.374] with a width of 0.020 (**Supplementary Table 3** and **Supplementary Table 4** in the **Supplementary Tables**, **Supplementary Figure 8** in the **Supplementary Figures**). Regarding the impact of prompting strategies, for the DKD diagnosis task, simple queries (Prompt 1) or prompts with more explicit instructions (Prompt 2) generally performed better. It was noteworthy that Prompt 4, which introduced Chain-of-Thought (CoT), did not perform optimally in this task; especially under Chinese prompts (**Figure 4B** and **Supplementary Figure 16** in the **Supplementary Figures**).

#### Task 3: metabolic syndrome diagnosis

For the metabolic syndrome diagnosis task, models like Qwen2-2B, the Llama series, and Gemma2-9B showed commendable stability, with consistent confidence interval widths of 0.019–0.022 under multiple prompts. The DeepSeek-32B model, when combined with Chinese Prompt 3, achieved the highest F1-score of 0.650 (95% CI, 0.638–0.660) (**Supplementary Table 5** and **Supplementary Table 6** in the **Supplementary Tables**, **Supplementary Figure 9** in the **Supplementary Figures**). Regarding prompting, Prompt 4 was generally superior, improving performance for smaller or reasoning-sensitive models (e.g., MMedLM2-7B). Conversely, its impact was less effective for models with strong inherent generalization or insensitivity to CoT, such as Qwen2.5-14B-int4 (**Figure 4**C and **Supplementary Figure 17** in the **Supplementary Figures**).

### Impact of model characteristics

#### Model size

We investigated the relationship between model parameter scale and diagnostic performance in the Qwen and DeepSeek series. A general upward trend in F1 scores with increasing size confirmed enhanced capabilities in larger models. However, this scaling was not linear. In the Qwen series, performance peaked at the 32B scale and then slightly declined at 72B (**Supplementary Figure 10A** in the **Supplementary Figures**). For the DeepSeek series, the 671B model (DeepSeek-R1) showed only a marginal advantage over the smaller 32B distilled version. Notably, the 671B model’s average F1-score was even slightly lower than the 32B model’s under English prompts, indicating that advanced distillation can yield competitive performance in smaller models (**Supplementary Figure 10B** in the **Supplementary Figures**).

#### Model family

When comparing the performance of different model families, we focused on the performance of the Qwen series and DeepSeek series in Task 1.By calculating the average and optimal values of the average Weighted F1 scores for these two-model series under Chinese (zh) and English (eng) prompts, the results showed that although the Qwen model family demonstrated superior performance on average, as measured by weighted F1 scores across Chinese and English prompts, the optimal scores for both series were closely matched (**Supplementary Figure 11** in the **Supplementary Figures**).

#### General vs. medical models

We compared general-purpose (Qwen, Llama and Gemma) and medically fine-tuned (HuatuoGPT, MMedLM2) models at the ~7B scale, revealing a strong task-dependent effect. In the broad diabetes classification task, the general-purpose Qwen2.5-7B showed exceptional bilingual performance (mean F1: ~0.93), outperforming medical models. While HuatuoGPT-o1-7B also performed strongly (mean F1: ~0.91-0.92), another medical model, MMedLM2-7B, exhibited poor English generalization (F1: 0.697), highlighting inconsistency (**Supplementary Figure 12A** in the **Supplementary Figures**). Conversely, for the specialized DKD and MetS tasks, HuatuoGPT-o1-7B demonstrated a clear advantage, surpassing even larger general-purpose models. In contrast, other medical models like HuatuoGPT2 and MMedLM2 performed poorly on these tasks, particularly under English prompts, suggesting issues with their inherent capabilities or our secondary evaluation strategy (**Supplementary Figures 12B, C** in the **Supplementary Figures**).

#### Quantization

To evaluate the impact of INT4 quantization on model performance, we compared the Qwen2.5-14B and Llama-3.2-11B models and their corresponding INT4 quantized versions. The results showed that INT4 quantization of these models led to a slight decrease in overall performance, but they generally maintained high usability. Specifically, the INT4 quantized version of Qwen2.5-14B experienced an average F1 score decrease of 0.002 and 0.006 on Chinese and English prompts, respectively, with an overall average F1 score decrease of 0.004. For the INT4 quantized version of Llama-3.2-11B, the performance decrease was slightly larger, with the average F1 score decreasing by about 0.011 on Chinese tasks, and increasing by 0.005 on English tasks, resulting in an overall average F1 score decrease of 0.003. Although there were minor differences in performance, these decreases were generally within acceptable limits (**Supplementary Figure 13** in the **Supplementary Figures**).

#### Distillation

We investigated the impact of knowledge distillation techniques on model performance, specifically comparing the performance of distilled DeepSeek series models (DS-R1-Distill-Qwen) with the original Qwen 2.5 models of corresponding parameter scales. The distilled DeepSeek models, in most cases, did not outperform their corresponding original Qwen models of the same parameter scale. At the 14B and 32B parameter levels, the distilled DS-R1-Distill-Qwen models in Chinese and English prompts generally had similar average F1 scores compared to the corresponding Qwen2.5 models. But at 7B parameter level, the distilled DS-R1-Distill-Qwen model was substantially lower than that of the corresponding Qwen2.5 model (**Supplementary Figure 14** in the **Supplementary Figures**).

### Prompting strategy

Our evaluation of four prompting strategies revealed that their effectiveness varied significantly across tasks. For diabetes classification (Task 1), while CoT (Prompt 4) showed potential for minority class recall, the simplest prompt (Prompt 1) achieved the highest average macro F1-score, with English prompts generally outperforming Chinese ones (**Figure 4A** and **Supplementary Figure 15** in the **Supplementary Figures**). In DKD diagnosis (Task 2), simpler queries (Prompts 1 & 2) were more effective; CoT was largely detrimental, especially in English prompts (**Figure 4**B and **Supplementary Figure 16** in the **Supplementary Figures**). In contrast, for MetS diagnosis (Task 3), CoT (Prompt 4) was superior, yielding the best average F1-scores in both languages. It significantly boosted performance for smaller or reasoning-sensitive models, but its effect was less pronounced for models with strong inherent generalization (**Figure 4C** and **Supplementary Figure 17** in the **Supplementary Figures**).

### Qualitative error analysis

To precisely pinpoint the failure loci in rule-based diagnostic tasks, we conducted a supplementary structured-output experiment using DeepSeek-R1. The model was mandated to explicitly extract key diagnostic criteria alongside its final predictions for DKD and MetS, enabling a traceable audit of its reasoning process. Our analysis revealed that severe clinical data absence was a primary driver of diagnostic failure. In the DKD task, the overall missing rates for UACR and AER were 64.19% and 59.36%, respectively. Specifically, within incorrectly predicted cases, UACR and AER were missing in 52.01% and 46.09% of instances, with both completely absent in 24.13% of failures (**Supplementary Figure 18A**). While this widespread missingness frequently forced the model into a default “conservative” negative prediction, the explicit extraction exposed a critical “hallucination” vulnerability: in 0.83% of cases where diagnostic indicators were entirely absent, the model still baselessly generated a positive diagnosis. Furthermore, even when at least one indicator was successfully extracted, the diagnostic error rate remained at 27.02%. The audit further exposed inherent logical inconsistencies in complex rule execution. For the MetS task, significant missing rates persisted among incorrectly predicted cases. Beyond data absence, the model demonstrated severe reasoning disconnects during threshold integration and conditional counting: 49.77% of cases where the model explicitly extracted and self-identified meeting ≥3 criteria were still misclassified. Conversely, 21.94% of cases with <3 extracted criteria incorrectly received positive predictions (**Supplementary Figure 18B**). Ultimately, these findings demonstrate that while pervasive real-world data incompleteness heavily restricts rule-based LLM performance, the models also suffer from intrinsic reasoning biases and hallucinations when navigating complex, multi-conditional clinical logic.

## Discussion

Our systematic evaluation of open-source large language models (LLMs) on diagnostic tasks from 11,329 real-world patient records revealed a key paradox: models excelled at the cognitively demanding, multi-class task of diabetes subtyping but faltered on the seemingly simpler, rule-based binary diagnoses of diabetic kidney disease (DKD) and metabolic syndrome (MetS). This principal finding suggested that LLM performance in the clinical domain was dictated not by superficial task simplicity but by a sophisticated interplay between the nature of the required clinical reasoning, model architecture, and prompting strategy.

A pivotal finding was the models’ superior performance on diabetes subtyping, a task that necessitated synthesizing sparse, heterogeneous data from unstructured clinical narratives. The high fidelity achieved, particularly by the DeepSeek series, indicated a robust capacity for high-dimensional pattern recognition, a departure from rigid, rule-based execution. This successful translation of performance from standardized benchmarks to the ecologically valid challenge of processing noisy, real-world clinical text marked a significant step toward clinical implementation. Conversely, the models exhibited brittle performance on DKD and MetS tasks, revealing a core limitation in executing procedural rules that demand strict quantitative and logical adherence. This deficit, analogous to the “factual hallucination” and “computational weakness” observed in other domains (4, 24, 25), suggested that the probabilistic nature of LLMs inhibited their function as reliable “rule engines,” a limitation that was particularly acute when diagnostic criteria were fragmented across clinical text.

Our results underscored that the utility of prompt engineering was highly task dependent. For the complex diabetes subtyping, Chain-of-Thought (CoT) prompting enhanced the identification of minority classes by structuring the model’s pattern-matching process, consistent with prior work (26). For MetS diagnosis, CoT’s value derived from its ability to decompose a complex rule set into a verifiable checklist, thereby promoting “faithful reasoning” (27, 28). In contrast, the superiority of simpler prompts for DKD diagnosis suggested that tasks involving complex exclusionary logic remain a significant challenge for LLMs, where excessive guidance may paradoxically increase the risk of logical fallacies (29–31).

Our investigation into model characteristics yielded several nuanced insights. First, the “bigger is better” paradigm exhibited non-linear scaling effects, with performance plateauing around the 32B parameter scale, aligning with observations of diminishing returns at massive scales (32). Second, the trade-off between general-purpose and medically specialized models was evident. While generalist models demonstrated robust reasoning, our findings, in line with studies on models like HuatuoGPT, affirmed that domain-specific fine-tuning provided a critical advantage for certain nuanced tasks, suggesting a hybrid approach might be optimal (33).

Furthermore, for clinical deployment, our findings on model compression were particularly relevant. The negligible performance penalty from INT4 quantization confirmed its viability for resource-constrained environments, consistent with research on QLoRA and GPTQ (34, 35). In contrast, the inconsistent efficacy of knowledge distillation highlighted its complexity, suggesting that simple distillation might be insufficient for multifaceted clinical reasoning tasks (36, 37).

A consistent and critical finding was the superior performance of English prompts on Chinese clinical text, highlighting the Anglocentric bias of many foundational models. This aligned with large-scale multilingual evaluations (38) and underscored an urgent need for developing high-quality, non-English medical LLMs and advancing cross-lingual transfer learning to ensure AI fairness and inclusivity in global health (21, 39).

The findings from our qualitative error analysis provide critical insights into the limitations of applying rigid rule-based LLM frameworks to real-world clinical data. By designing a structured evaluation that required the models to explicitly extract key clinical indicators alongside the final diagnosis, we demonstrated that the models’ underperformance in identifying specific diagnostic criteria for comorbidities is not strictly an algorithmic reasoning deficit but is heavily a consequence of data absence and extraction challenges within complex, unstructured narratives. This highlights a fundamental reality in clinical informatics: while forcing LLMs to evaluate each explicit diagnostic criterion exposes the brittleness of rule-based logic when confronted with missing values, it simultaneously underscores the necessity of holistic contextual synthesis. Ultimately, improving LLM utility in specialized fields like endocrinology will require advancing not only their deductive logic capabilities but also developing robust computational strategies to handle the pervasive data incompleteness characteristic of real-world medical records (23, 40).

The qualitative analysis demonstrates that guiding LLMs with criteria-driven prompts significantly improves their diagnostic extraction for complex conditions. This aligns seamlessly with recent findings by Washington et al. (41), who demonstrated the clear efficacy of using clinical scoresheets to facilitate LLM diagnostic classification from unstructured text. By decomposing complex diagnostic rule sets into structured, step-by-step checklists, LLMs can more effectively navigate clinical narratives, thereby reducing hallucinations and significantly improving the transparency and traceability of their clinical reasoning.

Interestingly, our results indicate that generalist models sometimes outperform medical-specific models in holistic, semantic tasks like complex diabetes subtyping. However, this observation does not diminish the value of domain-specific adaptation; rather, it highlights that the advantage of specialized models is highly task-dependent. For instance, Abdur et al. (42) demonstrated the significant benefits of domain-specific fine-tuning by developing “nBERT” specifically for emotion recognition in psychotherapy. While large generalist models excel at broad semantic reasoning in our holistic subtyping tasks, specialized fine-tuning remains a crucial and powerful strategy for extracting highly nuanced, domain-specific clinical features where off-the-shelf generalist capabilities may lack precision.

Our study had several key strengths. First, its foundation in real-world, unstructured Chinese clinical narratives provided a realistic testbed for LLM utility, complementing studies that relied on standardized datasets (4). Second, our in-depth, domain-specific analysis of endocrinological reasoning offered a granular perspective that complements broad evaluation frameworks like the BRIDGE Benchmark (7). Third, our focus on the open-source ecosystem provided actionable insights for developing reproducible and accessible clinical AI solutions. Despite these strengths, several limitations need to be acknowledged. The retrospective, single-center design might limit the generalizability of our findings. Additionally, the use of automated methods for ground-truth labeling introduced a potential for bias, and the data was confined to discharge summaries, omitting other valuable clinical data sources. Specifically, evaluating complex comorbidities like DKD and MetS as binary classifications from single-encounter summaries ignores inherent temporal and missing data challenges, such as absent longitudinal eGFR changes or missing laboratory values required for MetS criteria. Therefore, the observed failure of rule-based reasoning in these specific tasks is partly attributable to real-world data incompleteness rather than purely an algorithmic reasoning deficit. Future multi-center, prospective studies incorporating multi-modal data were warranted to validate and extend these findings.

## Conclusion

In conclusion, LLMs demonstrated a fundamental trade-off between holistic pattern recognition and procedural fidelity. Their success in complex classifications and failure in rule-based tasks underscored that their core strength was contextual synthesis, not logical reasoning. This transformed them from inscrutable black boxes into optimizable systems. These findings suggest that rather than functioning as autonomous diagnostic agents, the most appropriate future application of these models may be as “clinical co-pilots,” augmenting expert decision-making. However, because this study focused on computational evaluation without human-in-the-loop experiments, rigorous clinical trials involving direct clinician interaction are necessary to formally test and validate their efficacy and safety in this collaborative role.

## Data Availability

Due to the sensitive nature of the patient information contained in the unstructured clinical notes, the raw data used in this study are confidential and cannot be shared to protect patient privacy. Requests to access the datasets should be directed to Zhiguang Zhou, zhouzhiguang@csu.edu.cn.
