# Supplementary material for: Real-world performance of open-source large language models in diabetes diagnosis
Source: Front Endocrinol (Lausanne). 2026 Mar 25;17:1747468. doi: 10.3389/fendo.2026.1747468 (PMC13056687; doi:10.3389/fendo.2026.1747468)
Supplement: Supplementary file 1 [file DataSheet1.docx]

**Supplemental appendix tables**

**Title:** Real-World Performance of Open-Source Large Language Models in Diabetes Diagnosis

**Table S1**. Performance comparison of large language models on diabetes classification diagnosis task (Task 1).

**Table S2.** The 95%CI of Weighted F1 scores of large language models on diabetes classification diagnosis task (Task 1).

**Table S3.** Performance comparison of large language models on diabetes kidney disease (DKD) diagnosis task (Task 2).

**Table S4**. The 95%CI of F1 scores of large language models on diabetic kidney disease diagnosis task (Task 2).

**Table S5.** Performance comparison of large language models on metabolic syndrome diagnosis task (Task 3).

**Table S6.** The 95%CI of F1 scores of large language models on metabolic syndrome diagnosis task (Task 3).

**Table S1. Performance comparison of large language models on diabetes classification diagnosis task (Task 1).**

| **Task 1: Diabetes Classification Diagnosis (Accuracy)** | | | | | | | | | |
| --- | --- | --- | --- | --- | --- | --- | --- | --- | --- |
| Series | Models | Chinese Prompt | | | | English Prompt | | | |
|  |  | Prompt 1 | Prompt 2 | Prompt 3 | Prompt 4 | Prompt 1 | Prompt 2 | Prompt 3 | Prompt 4 |
| DeepSeek LLMs | DS-R1-Distill-Qwen-32B | 93.02% | 90.51% | 91.60% | 92.11% | 93.45% | 92.98% | 93.18% | 93.72% |
|  | DS-R1-Distill-Qwen-14B | 85.42% | 81.99% | 87.65% | 86.93% | 91.76% | 91.97% | 92.13% | 92.02% |
|  | DS-R1-Distill-Qwen-7B | 82.27% | 74.67% | 63.27% | 63.22% | 89.02% | 85.15% | 82.64% | 82.68% |
|  | DeepSeek-Distill-32B | 92.39% | 90.59% | 92.68% | 92.33% | 94.40% | 93.93% | 93.62% | 93.73% |
|  | DeepSeek-R1 | 93.33% | 91.87% | 92.03% | 91.85% | 92.74% | 91.47% | 92.36% | 93.36% |
|  | DeepSeek-V3 | **95.21%** | 92.53% | 92.61% | 92.59% | 94.95% | 92.06% | 93.94% | 94.98% |
| Qwen LLMs | QwQ-32B | 94.12% | 92.87% | 90.90% | 89.10% | 94.24% | 92.81% | 92.73% | 92.83% |
|  | Qwen2.5-72B | 94.77% | 94.66% | 94.10% | 94.28% | 94.77% | 94.71% | 93.85% | 93.99% |
|  | Qwen2.5-32B | 94.77% | 94.58% | 93.80% | 94.61% | 94.86% | 94.44% | 94.90% | 94.69% |
|  | Qwen2.5-14B | 94.53% | 94.07% | 93.94% | 92.20% | 94.50% | 94.09% | 93.66% | 93.67% |
|  | Qwen2.5-7B | 93.53% | 93.10% | 92.98% | 93.61% | 94.30% | 94.20% | 93.64% | 94.11% |
|  | Qwen2.5-14B-int4 | 94.17% | 94.07% | 93.96% | 93.48% | 94.16% | 94.09% | 92.89% | 93.17% |
|  | Qwen2-2B | 91.29% | 90.61% | 90.80% | 90.44% | 91.83% | 91.57% | 90.49% | 90.70% |
| Llama LLMs | Llama-3.2-11B | 92.75% | 91.75% | 90.18% | 91.94% | 80.54% | 79.06% | 69.24% | 91.69% |
|  | Llama-3.2-11B-int4 | 91.92% | 90.83% | 91.66% | 91.46% | 85.30% | 73.13% | 75.64% | 91.06% |
| Other LLMs | Gemma2-9B | 93.43% | 92.99% | 92.85% | 92.19% | 93.36% | 92.05% | 58.65% | 92.27% |
|  | Apollo-7B | 83.68% | 84.67% | 74.30% | 61.09% | 65.56% | 68.42% | 36.50% | 43.84% |
| Medical Models | MMedLM2-7B | 85.66% | 93.15% | 88.86% | 92.80% | 73.22% | 62.93% | 32.22% | 91.24% |
|  | huatuoGPT-o1-7B | 93.39% | 92.78% | 92.41% | 92.86% | 91.19% | 92.39% | 89.92% | 92.50% |
|  | huatuoGPT2-7B | 89.68% | 83.14% | 88.09% | 87.48% | 92.21% | 86.61% | 86.08% | 90.70% |
| **Task 1: Diabetes Classification Diagnosis (Weighted F1)** | | | | | | | | | |
| Series | Models | Chinese Prompt | | | | English Prompt | | | |
|  |  | Prompt 1 | Prompt 2 | Prompt 3 | Prompt 4 | Prompt 1 | Prompt 2 | Prompt 3 | Prompt 4 |
| DeepSeek LLMs | DS-R1-Distill-Qwen-32B | 0.93426 | 0.91952 | 0.92195 | 0.92676 | 0.93798 | 0.93537 | 0.93407 | 0.93750 |
|  | DS-R1-Distill-Qwen-14B | 0.88519 | 0.86728 | 0.89917 | 0.89379 | 0.92789 | 0.92741 | 0.92831 | 0.92686 |
|  | DS-R1-Distill-Qwen-7B | 0.83803 | 0.79455 | 0.72402 | 0.72097 | 0.87649 | 0.85563 | 0.84405 | 0.84235 |
|  | DeepSeek-Distill-32B | 0.92964 | 0.91871 | 0.93163 | 0.93031 | 0.94356 | 0.94109 | 0.93755 | 0.93729 |
|  | DeepSeek-R1 | 0.93926 | 0.93187 | 0.92851 | 0.92900 | 0.93762 | 0.93052 | 0.93312 | 0.93593 |
|  | DeepSeek-V3 | **0.95127** | 0.93468 | 0.93432 | 0.93373 | 0.94811 | 0.93238 | 0.94228 | 0.94723 |
| Qwen LLMs | QwQ-32B | 0.94488 | 0.93698 | 0.92752 | 0.91562 | 0.94572 | 0.93654 | 0.93596 | 0.93537 |
|  | Qwen2.5-72B | 0.93779 | 0.93614 | 0.92842 | 0.93006 | 0.93738 | 0.93664 | 0.92358 | 0.92592 |
|  | Qwen2.5-32B | 0.94466 | 0.94382 | 0.93791 | 0.94266 | 0.94369 | 0.94242 | 0.94364 | 0.94160 |
|  | Qwen2.5-14B | 0.93986 | 0.93607 | 0.93349 | 0.92556 | 0.93837 | 0.93080 | 0.92779 | 0.92779 |
|  | Qwen2.5-7B | 0.92995 | 0.92725 | 0.92491 | 0.93097 | 0.93187 | 0.93004 | 0.92935 | 0.93036 |
|  | Qwen2-2B | 0.88034 | 0.86551 | 0.86988 | 0.86153 | 0.89110 | 0.88873 | 0.86268 | 0.86760 |
|  | Qwen2.5-14B-int4 | 0.93533 | 0.92974 | 0.93196 | 0.93078 | 0.93271 | 0.92890 | 0.92080 | 0.91733 |
| Llama LLMs | Llama-3.2-11B | 0.90690 | 0.88758 | 0.88869 | 0.89198 | 0.85747 | 0.84711 | 0.78141 | 0.90510 |
|  | Llama-3.2-11B-int4 | 0.89081 | 0.86766 | 0.89023 | 0.88142 | 0.87949 | 0.80958 | 0.82087 | 0.90108 |
| Other LLMs | Gemma2-9B | 0.92171 | 0.91219 | 0.91095 | 0.89994 | 0.92762 | 0.91572 | 0.70344 | 0.91424 |
|  | Apollo-7B | 0.86204 | 0.86132 | 0.79985 | 0.71874 | 0.75419 | 0.77255 | 0.51775 | 0.58438 |
| Medical Models | MMedLM2-7B | 0.86570 | 0.91760 | 0.88861 | 0.90664 | 0.78463 | 0.70779 | 0.40221 | 0.89473 |
|  | huatuoGPT-o1-7B | 0.92886 | 0.92263 | 0.91633 | 0.91114 | 0.91146 | 0.91952 | 0.89878 | 0.91435 |
|  | huatuoGPT2-7B | 0.88803 | 0.85349 | 0.87216 | 0.86734 | 0.91053 | 0.87695 | 0.85627 | 0.87842 |
| **Task 1: Diabetes Classification Diagnosis (Macro F1)** | | | | | | | | | |
| Series | Models | Chinese Prompt | | | | English Prompt | | | |
|  |  | Prompt 1 | Prompt 2 | Prompt 3 | Prompt 4 | Prompt 1 | Prompt 2 | Prompt 3 | Prompt 4 |
| DeepSeek LLMs | DS-R1-Distill-Qwen-32B | 0.66495 | 0.59745 | 0.61208 | 0.65205 | 0.65782 | 0.64556 | 0.63958 | 0.70030 |
|  | DS-R1-Distill-Qwen-14B | 0.54667 | 0.53176 | 0.59359 | 0.58183 | 0.63241 | 0.62198 | 0.63220 | 0.64752 |
|  | DS-R1-Distill-Qwen-7B | 0.36502 | 0.32625 | 0.33453 | 0.31645 | 0.41204 | 0.39367 | 0.43278 | 0.40880 |
|  | DeepSeek-still-32B | 0.65108 | 0.64192 | 0.64519 | 0.68432 | 0.67592 | 0.67923 | 0.65431 | 0.68414 |
|  | DeepSeek-R1 | 0.68395 | 0.65420 | 0.64346 | 0.67309 | 0.66783 | 0.63983 | 0.65261 | 0.67056 |
|  | DeepSeek-V3 | 0.68518 | 0.62851 | 0.67643 | 0.68903 | 0.71110 | 0.63774 | 0.65814 | **0.72983** |
| Qwen LLMs | QwQ-32B | 0.71356 | 0.71811 | 0.66934 | 0.66515 | 0.71589 | 0.69492 | 0.64955 | 0.67844 |
|  | Qwen2.5-72B | 0.61524 | 0.60865 | 0.58158 | 0.59287 | 0.62095 | 0.62064 | 0.58170 | 0.58158 |
|  | Qwen2.5-32B | 0.70377 | 0.70262 | 0.66485 | 0.70905 | 0.68101 | 0.68630 | 0.67648 | 0.66339 |
|  | Qwen2.5-14B | 0.68414 | 0.67219 | 0.65321 | 0.65254 | 0.65592 | 0.60781 | 0.64220 | 0.65009 |
|  | Qwen2.5-7B | 0.58519 | 0.58826 | 0.60814 | 0.65476 | 0.57273 | 0.52962 | 0.60200 | 0.61186 |
|  | Qwen2-2B | 0.29293 | 0.25316 | 0.26507 | 0.24212 | 0.32138 | 0.31657 | 0.24540 | 0.25887 |
|  | Qwen2.5-14B-int4 | 0.65027 | 0.59926 | 0.65354 | 0.66003 | 0.60720 | 0.58946 | 0.60173 | 0.59417 |
| Llama LLMs | Llama-3.2-11B | 0.38220 | 0.32063 | 0.46378 | 0.44212 | 0.55031 | 0.53126 | 0.48421 | 0.56555 |
|  | Llama-3.2-11B-int4 | 0.31861 | 0.25685 | 0.35931 | 0.38364 | 0.52725 | 0.50467 | 0.50353 | 0.54843 |
| Other LLMs | Gemma2-9B | 0.54196 | 0.50797 | 0.51220 | 0.51242 | 0.62060 | 0.59240 | 0.40780 | 0.57627 |
|  | Apollo-7B | 0.42470 | 0.34562 | 0.34819 | 0.32583 | 0.38550 | 0.32591 | 0.30570 | 0.27773 |
| Medical Models | MMedLM2-7B | 0.33659 | 0.39228 | 0.35700 | 0.36205 | 0.32506 | 0.26113 | 0.15243 | 0.34284 |
|  | huatuoGPT-o1-7B | 0.61209 | 0.56771 | 0.46680 | 0.53952 | 0.53874 | 0.53834 | 0.48887 | 0.54211 |
|  | huatuoGPT2-7B | 0.35054 | 0.33650 | 0.32262 | 0.35860 | 0.38898 | 0.36673 | 0.30782 | 0.30306 |

**Note:** The performance comparison metrics (including Accuracy, Weighted F1 score, Marco F1-score) of the algorithm models on **Task 1**. Each row represents a model, and each column represents an evaluation metric. Values in **bold** denote the best-performing results.

**Table S2. The 95%CI of Weighted F1 scores of large language models on diabetes classification diagnosis task (Task 1).**

| Series | Models | Chinese Prompt | | | | English Prompt | | | |
| --- | --- | --- | --- | --- | --- | --- | --- | --- | --- |
|  |  | Prompt1 | Prompt2 | Prompt3 | Prompt4 | Prompt1 | Predict 2 | Prompt3 | Prompt4 |
| DeepSeek LLMs | DS-R1-Distill-Qwen-32B | [0.930, 0.939] | [0.914, 0.925] | [0.916, 0.927] | [0.922, 0.932] | [0.933, 0.943] | [0.930, 0.940] | [0.929, 0.939] | [0.932, 0.942] |
|  | DS-R1-Distill-Qwen-14B | [0.879, 0.891] | [0.861, 0.873] | [0.893, 0.905] | [0.888, 0.900] | [0.923, 0.933] | [0.922, 0.932] | [0.923, 0.933] | [0.922, 0.932] |
|  | DS-R1-Distill-Qwen-7B | [0.830, 0.846] | [0.786, 0.802] | [0.715, 0.732] | [0.713, 0.729] | [0.869, 0.884] | [0.848, 0.863] | [0.837, 0.851] | [0.835, 0.850] |
|  | DeepSeek-Distill-32B | [0.925, 0.934] | [0.914, 0.924] | [0.927, 0.937] | [0.925, 0.935] | [0.939, 0.948] | [0.936, 0.946] | [0.933, 0.942] | [0.932, 0.942] |
|  | DeepSeek-R1 | [0.935, 0.944] | [0.927, 0.937] | [0.923, 0.933] | [0.924, 0.933] | [0.933, 0.942] | [0.925, 0.935] | [0.929, 0.938] | [0.931, 0.941] |
|  | DeepSeek-V3 | **[0.947, 0.955]** | [0.930, 0.939] | [0.930, 0.939] | [0.929, 0.939] | [0.944, 0.953] | [0.928, 0.937] | [0.937, 0.947] | [0.942, 0.952] |
| Qwen LLMs | QwQ-32B | [0.941, 0.949] | [0.932, 0.941] | [0.923, 0.932] | [0.911, 0.920] | [0.942, 0.950] | [0.932, 0.941] | [0.931, 0.940] | [0.931, 0.940] |
|  | Qwen2.5-72B | [0.932, 0.943] | [0.931, 0.942] | [0.922, 0.934] | [0.924, 0.936] | [0.932, 0.943] | [0.931, 0.942] | [0.917, 0.930] | [0.920, 0.932] |
|  | Qwen2.5-32B | [0.940, 0.950] | [0.939, 0.949] | [0.933, 0.943] | [0.938, 0.947] | [0.939, 0.948] | [0.938, 0.947] | [0.939, 0.949] | [0.937, 0.946] |
|  | Qwen2.5-14B | [0.934, 0.945] | [0.931, 0.941] | [0.928, 0.939] | [0.920, 0.931] | [0.933, 0.944] | [0.925, 0.936] | [0.922, 0.934] | [0.922, 0.933] |
|  | Qwen2.5-7B | [0.924, 0.935] | [0.922, 0.933] | [0.919, 0.930] | [0.925, 0.936] | [0.926, 0.938] | [0.924, 0.936] | [0.923, 0.935] | [0.924, 0.936] |
|  | Qwen2.5-14B-int4 | [0.930, 0.941] | [0.923, 0.936] | [0.926, 0.937] | [0.925, 0.936] | [0.927, 0.938] | [0.923, 0.935] | [0.915, 0.927] | [0.910, 0.924] |
|  | Qwen2-2B | [0.872, 0.888] | [0.857, 0.874] | [0.862, 0.878] | [0.852, 0.870] | [0.883, 0.899] | [0.881, 0.897] | [0.855, 0.871] | [0.859, 0.876] |
| Llama LLMs | Llama-3.2-11B | [0.900, 0.914] | [0.880, 0.895] | [0.882, 0.896] | [0.884, 0.900] | [0.851, 0.864] | [0.840, 0.854] | [0.773, 0.789] | [0.898, 0.911] |
|  | Llama-3.2-11B-int4 | [0.883, 0.899] | [0.860, 0.876] | [0.882, 0.898] | [0.873, 0.889] | [0.873, 0.886] | [0.802, 0.817] | [0.814, 0.828] | [0.894, 0.908] |
| Other LLMs | Gemma2-9B | [0.915, 0.928] | [0.905, 0.919] | [0.905, 0.918] | [0.892, 0.908] | [0.922, 0.933] | [0.910, 0.922] | [0.695, 0.712] | [0.908, 0.920] |
|  | Apollo-7B | [0.855, 0.870] | [0.854, 0.869] | [0.793, 0.807] | [0.711, 0.727] | [0.746, 0.762] | [0.764, 0.780] | [0.507, 0.527] | [0.575, 0.594] |
| Medical Models | MMedLM2-7B | [0.859, 0.872] | [0.911, 0.924] | [0.882, 0.895] | [0.900, 0.913] | [0.777, 0.792] | [0.699, 0.716] | [0.392, 0.413] | [0.888, 0.902] |
|  | huatuoGPT-o1-7B | [0.923, 0.934] | [0.917, 0.928] | [0.910, 0.922] | [0.904, 0.918] | [0.906, 0.917] | [0.914, 0.925] | [0.893, 0.905] | [0.908, 0.920] |
|  | huatuoGPT2-7B | [0.881, 0.895] | [0.846, 0.861] | [0.865, 0.880] | [0.860, 0.875] | [0.904, 0.917] | [0.870, 0.883] | [0.848, 0.864] | [0.870, 0.886] |

**Notes:** All values represented the 95% confidence intervals (CIs) for weighted F1 scores. DeepSeek-V3, when paired with Chinese prompt 1(**bold noted**), exhibited the narrowest interval and the highest upper bound.

**Table S3. Performance comparison of large language models on diabetes kidney disease (DKD) diagnosis task (Task 2).**

| **Task 2: Diabetic Kidney Disease Diagnosis (Accuracy)** | | | | | | | | | |
| --- | --- | --- | --- | --- | --- | --- | --- | --- | --- |
| Series | Models | Chinese Prompt | | | | English Prompt | | | |
|  |  | Prompt 1 | Prompt 2 | Prompt 3 | Prompt 4 | Prompt 1 | Prompt 2 | Prompt 3 | Prompt 4 |
| Qwen LLMs | Qwen2-2B | 52.58% | 28.98% | 55.55% | 26.85% | 73.48% | 37.26% | 65.67% | 23.97% |
|  | Qwen2.5-14B-int4 | 64.81% | 62.00% | 50.23% | 54.42% | 77.52% | 67.28% | 57.93% | 68.81% |
| Llama LLMs | Llama-3.2-11B | 35.20% | 44.42% | 62.99% | 40.37% | 48.37% | 63.62% | 70.00% | 73.02% |
|  | Llama-3.2-11B-int4 | 30.95% | 40.03% | 55.07% | 56.28% | 41.81% | 54.57% | 57.45% | 57.93% |
| Gemma LLM | Gemma2-9B | 44.39% | 44.89% | 39.03% | 43.54% | 52.56% | 50.79% | 35.12% | 58.58% |
| Medical Models | huatuoGPT-o1-7B | 57.46% | 61.28% | 60.84% | 53.39% | 59.07% | 66.10% | 62.48% | 70.64% |
|  | huatuoGPT2-7B (Twice) | 66.22% | 52.48% | 64.45% | 63.95% | 69.81% | 77.60% | 73.33% | 69.47% |
|  | MMedLM2-7B (Twice) | 78.64% | 78.53% | 74.86% | 70.34% | 78.13% | 78.17% | 77.74% | 77.33% |
| DeepSeek LLM | DeepSeek-R1 | 72.05% | 74.05% | 68.94% | 72.23% | **79.40%** | 72.42% | 71.07% | 75.01% |
| **Task 2: Diabetic Kidney Disease Diagnosis (Recall/Sensitivity)** | | | | | | | | | |
| Series | Models | Chinese Prompt | | | | English Prompt | | | |
|  |  | Prompt 1 | Prompt 2 | Prompt 3 | Prompt 4 | Prompt 1 | Prompt 2 | Prompt 3 | Prompt 4 |
| Qwen LLMs | Qwen2-2B | 0.81466 | 0.96883 | 0.74810 | 0.95619 | 0.64869 | 0.93050 | 0.59773 | **0.98273** |
|  | Qwen2.5-14B-int4 | 0.86394 | 0.86057 | 0.90564 | 0.87658 | 0.68618 | 0.82098 | 0.84751 | 0.78222 |
| Llama LLMs | Llama-3.2-11B | 0.95661 | 0.88711 | 0.71272 | 0.90691 | 0.88416 | 0.72620 | 0.56445 | 0.34836 |
|  | Llama-3.2-11B-int4 | 0.96925 | 0.92165 | 0.81971 | 0.76074 | 0.92376 | 0.81592 | 0.76622 | 0.70135 |
| Gemma LLM | Gemma2-9B | 0.94419 | 0.92851 | 0.94332 | 0.89298 | 0.90609 | 0.88240 | 0.95770 | 0.82022 |
| Medical Models | huatuoGPT-o1-7B | 0.91112 | 0.85678 | 0.78939 | 0.86816 | 0.87577 | 0.81319 | 0.77520 | 0.70148 |
|  | huatuoGPT2-7B (Twice) | 0.44608 | 0.68618 | 0.38627 | 0.41491 | 0.24125 | 0.02527 | 0.15922 | 0.23547 |
|  | MMedLM2-7B (Twice) | 0.02583 | 0.03597 | 0.09013 | 0.11550 | 0.02514 | 0.02860 | 0.02641 | 0.03849 |
| DeepSeek LLM | DeepSeek-R1 | 0.86352 | 0.82056 | 0.81508 | 0.75906 | 0.36310 | 0.45198 | 0.41154 | 0.49789 |
| **Task 2: Diabetic Kidney Disease Diagnosis (Specificity)** | | | | | | | | | |
| Series | Models | Chinese Prompt | | | | English Prompt | | | |
|  |  | Prompt 1 | Prompt 2 | Prompt 3 | Prompt 4 | Prompt 1 | Prompt 2 | Prompt 3 | Prompt 4 |
| Qwen LLMs | Qwen2-2B | 0.44944 | 0.10951 | 0.50441 | 0.08627 | 0.75819 | 0.22461 | 0.67281 | 0.04235 |
|  | Qwen2.5-14B-int4 | 0.59084 | 0.55623 | 0.39542 | 0.45606 | 0.79877 | 0.63350 | 0.50821 | 0.66309 |
| Llama LLMs | Llama-3.2-11B | 0.19174 | 0.32674 | 0.60793 | 0.27024 | 0.37755 | 0.61228 | 0.73590 | 0.83149 |
|  | Llama-3.2-11B-int4 | 0.13456 | 0.26209 | 0.47940 | 0.51033 | 0.28409 | 0.47404 | 0.52373 | 0.54696 |
| Gemma LLM | Gemma2-9B | 0.31247 | 0.32313 | 0.24503 | 0.31548 | 0.42602 | 0.40997 | 0.19168 | 0.52510 |
| Medical Models | huatuoGPT-o1-7B | 0.48543 | 0.54807 | 0.56047 | 0.44534 | 0.53058 | 0.62310 | 0.58521 | 0.71057 |
|  | huatuoGPT2-7B (Twice) | 0.71949 | 0.48208 | 0.71301 | 0.69905 | 0.82079 | 0.97499 | 0.88572 | 0.81642 |
|  | MMedLM2-7B (Twice) | **0.99148** | 0.98922 | 0.94107 | 0.89107 | 0.98976 | 0.99143 | 0.98491 | 0.97361 |
| DeepSeek LLM | DeepSeek-R1 | 0.68252 | 0.71926 | 0.65606 | 0.71256 | 0.90821 | 0.79643 | 0.78995 | 0.81697 |
| **Task 2: Diabetic Kidney Disease Diagnosis (F1 Score)** | | | | | | | | | |
| Series | Models | Chinese Prompt | | | | English Prompt | | | |
|  |  | Prompt 1 | Prompt 2 | Prompt 3 | Prompt 4 | Prompt 1 | Prompt 2 | Prompt 3 | Prompt 4 |
| Qwen LLMs | Qwen2-2B | 0.41884 | 0.36384 | 0.41374 | 0.35411 | 0.50675 | 0.38344 | 0.42226 | 0.35143 |
|  | Qwen2.5-14B-int4 | 0.50711 | 0.48695 | 0.43268 | 0.44628 | 0.56124 | 0.51256 | 0.45779 | 0.51242 |
| Llama LLMs | Llama-3.2-11B | 0.38223 | 0.40080 | 0.44661 | 0.38926 | 0.41784 | 0.45548 | 0.44086 | 0.35117 |
|  | Llama-3.2-11B-int4 | 0.37038 | 0.39176 | 0.43331 | 0.42172 | 0.39953 | 0.42944 | 0.43013 | 0.41131 |
| Gemma LLM | Gemma2-9B | 0.41544 | 0.41357 | 0.39302 | 0.39838 | 0.44431 | 0.42882 | 0.38179 | 0.45351 |
| Medical Models | huatuoGPT-o1-7B | 0.47305 | 0.48114 | 0.45797 | 0.43842 | 0.47105 | 0.50059 | 0.46397 | 0.50212 |
|  | huatuoGPT2-7B (Twice) | 0.35627 | 0.37704 | 0.31292 | 0.32540 | 0.25171 | 0.04515 | 0.20026 | 0.24426 |
|  | MMedLM2-7B (Twice) | 0.04882 | 0.06682 | 0.13723 | 0.15106 | 0.04726 | 0.05390 | 0.04874 | 0.06766 |
| DeepSeek LLM | DeepSeek-R1 | 0.56419 | **0.56992** | 0.52375 | 0.53393 | 0.42484 | 0.40721 | 0.37347 | 0.45505 |

**Notes:** The performance comparison metrics (including Accuracy, Recall or sensitivity, Specificity and F1-score) of the algorithm models on Task 2. Each row represents a model, and each column represents an evaluation metric. Values in **bold** denote the best-performing results.

**Table S4. The 95%CI of F1 scores of large language models on diabetic kidney disease diagnosis task (Task 2).**

| **Task 2: Diabetic Kidney Disease Diagnosis (F1--95%CI)** | | | | | | | | | |
| --- | --- | --- | --- | --- | --- | --- | --- | --- | --- |
| Series | Models | Chinese Prompt | | | | English Prompt | | | |
|  |  | Prompt1 | Prompt2 | Prompt3 | Prompt4 | Prompt1 | Prompt2 | Prompt3 | Prompt4 |
| Qwen LLMs | Qwen2-2B | [0.406, 0.432] | [0.354, 0.374] | [0.401, 0.426] | [0.343, 0.365] | [0.491, 0.522] | [0.372, 0.395] | [0.407, 0.436] | [0.341, 0.362] |
|  | Qwen2.5-14B-int4 | [0.494, 0.520] | [0.474, 0.500] | [0.421, 0.444] | [0.433, 0.459] | [0.547, 0.577] | [0.499, 0.526] | [0.445, 0.471] | [0.499, 0.525] |
| Llama LLMs | Llama-3.2-11B | [0.371, 0.393] | [0.389, 0.413] | [0.433, 0.461] | [0.378, 0.400] | [0.406, 0.430] | [0.441, 0.468] | [0.426, 0.457] | [0.332, 0.368] |
|  | Llama-3.2-11B-int4 | [0.360, 0.381] | [0.381, 0.403] | [0.421, 0.446] | [0.408, 0.435] | [0.388, 0.410] | [0.417, 0.442] | [0.417, 0.443] | [0.399, 0.424] |
| Gemma LLM | Gemma2-9B | [0.404, 0.427] | [0.402, 0.426] | [0.381, 0.404] | [0.386, 0.410] | [0.432, 0.457] | [0.417, 0.441] | [0.370, 0.393] | [0.441, 0.467] |
| Medical Models | huatuoGPT-o1-7B | [0.460, 0.485] | [0.468, 0.494] | [0.445, 0.472] | [0.426, 0.450] | [0.458, 0.484] | [0.487, 0.513] | [0.450, 0.477] | [0.488, 0.517] |
|  | huatuoGPT2-7B (Twice) | [0.341, 0.371] | [0.364, 0.390] | [0.298, 0.329] | [0.312, 0.342] | [0.235, 0.268] | [0.034, 0.057] | [0.184, 0.217] | [0.226, 0.261] |
|  | MMedLM2-7B (Twice) | [0.037, 0.060] | [0.054, 0.081] | [0.121, 0.155] | [0.135, 0.167] | [0.036, 0.059] | [0.042, 0.067] | [0.038, 0.060] | [0.055, 0.081] |
| DeepSeek LLM | DeepSeek-R1 | [0.550, 0.577] | **[0.556, 0.583]** | [0.509, 0.537] | [0.520, 0.548] | [0.406, 0.442] | [0.388, 0.423] | [0.357, 0.389] | [0.438, 0.471] |

**Notes:** All values represented the 95% confidence intervals (CIs) for F1 scores. DeepSeek-R1, when paired with Chinese prompt 2 (bold noted), exhibited the narrowest interval and the highest upper bound.

**Table S5. Performance comparison of large language models on metabolic syndrome diagnosis task (Task 3).**

| **Task 3: Metabolic Syndrome Diagnosis (Accuracy)** | | | | | | | | | |
| --- | --- | --- | --- | --- | --- | --- | --- | --- | --- |
| Series | Models | Chinese Prompt | | | | English Prompt | | | |
|  |  | Prompt 1 | Prompt 2 | Prompt 3 | Prompt 4 | Prompt 1 | Prompt 2 | Prompt 3 | Prompt 4 |
| Qwen LLMs | Qwen2-2B | 33.40% | 33.20% | 33.36% | 33.22% | 54.01% | 42.43% | 33.60% | 33.19% |
|  | Qwen2.5-14B-int4 | 58.51% | 60.99% | 47.27% | 36.28% | 56.41% | 60.23% | 54.32% | 42.48% |
| Llama LLMs | Llama-3.2-11B | 39.47% | 38.44% | 42.12% | 42.97% | 38.49% | 44.90% | 43.95% | 65.17% |
|  | Llama-3.2-11B-int4 | 36.33% | 36.98% | 40.21% | 39.56% | 43.78% | 37.06% | 39.20% | 55.03% |
| Gemma LLM | Gemma2-9B | 36.31% | 36.83% | 37.04% | 40.32% | 39.13% | 39.50% | 43.11% | 36.74% |
| Medical Models | huatuoGPT-o1-7B | 66.56% | 70.95% | 73.85% | 70.72% | 67.58% | 72.82% | **74.22%** | 73.89% |
|  | huatuoGPT2-7B (Twice) | 65.52% | 64.94% | 64.32% | 65.91% | 64.58% | 61.44% | 64.06% | 57.16% |
|  | MMedLM2-7B (Twice) | 66.61% | 65.32% | 63.69% | 60.75% | 66.43% | 66.35% | 62.97% | 61.07% |
| DeepSeek LLMs | DeepSeek-32B | 66.48% | 66.85% | 70.64% | 66.10% | 64.42% | 66.30% | 66.69% | 66.03% |
|  | DeepSeek-R1 | 63.59% | 66.58% | 63.57% | 66.08% | 65.27% | 66.84% | 63.62% | 65.94% |
| **Task 3: Metabolic Syndrome Diagnosis (Recall / Sensitivity)** | | | | | | | | | |
| Series | Models | Chinese Prompt | | | | English Prompt | | | |
|  |  | Prompt 1 | Prompt 2 | Prompt 3 | Prompt 4 | Prompt 1 | Prompt 2 | Prompt 3 | Prompt 4 |
| Qwen LLMs | Qwen2-2B | 0.99947 | **0.99973** | **0.99973** | 0.99947 | 0.64135 | 0.87264 | 0.99893 | **0.99973** |
|  | Qwen2.5-14B-int4 | 0.93205 | 0.91687 | 0.97815 | 0.99627 | 0.92673 | 0.88542 | 0.92619 | 0.97176 |
| Llama LLMs | Llama-3.2-11B | 0.94698 | 0.94458 | 0.93099 | 0.95470 | 0.98614 | 0.91154 | 0.93072 | 0.46843 |
|  | Llama-3.2-11B-int4 | 0.97469 | 0.94431 | 0.96163 | 0.97629 | 0.90005 | 0.97362 | 0.97229 | 0.76072 |
| Gemma LLM | Gemma2-9B | 0.99306 | 0.98720 | 0.98666 | 0.97705 | 0.98453 | 0.97919 | 0.90878 | 0.99200 |
| Medical Models | huatuoGPT-o1-7B | 0.81680 | 0.65468 | 0.67653 | 0.77825 | 0.77736 | 0.58209 | 0.63876 | 0.57090 |
|  | huatuoGPT2-7B (Twice) | 0.06954 | 0.06930 | 0.18758 | 0.09435 | 0.14636 | 0.30667 | 0.14819 | 0.38810 |
|  | MMedLM2-7B (Twice) | 0.02239 | 0.03880 | 0.07826 | 0.22433 | 0.01390 | 0.01739 | 0.10929 | 0.13346 |
| DeepSeek LLMs | DeepSeek-32B | 0.88010 | 0.84466 | 0.82307 | 0.86144 | 0.86624 | 0.86757 | 0.83320 | 0.85532 |
|  | DeepSeek-R1 | 0.88862 | 0.86731 | 0.87663 | 0.86944 | 0.80256 | 0.58327 | 0.82094 | 0.85878 |
| **Task 3: Metabolic Syndrome Diagnosis (Specificity)** | | | | | | | | | |
| Series | Models | Chinese Prompt | | | | English Prompt | | | |
|  |  | Prompt 1 | Prompt 2 | Prompt 3 | Prompt 4 | Prompt 1 | Prompt 2 | Prompt 3 | Prompt 4 |
| Qwen LLMs | Qwen2-2B | 0.00436 | 0.00119 | 0.00356 | 0.00158 | 0.49003 | 0.20222 | 0.00766 | 0.00106 |
|  | Qwen2.5-14B-int4 | 0.41328 | 0.45789 | 0.22228 | 0.04897 | 0.38450 | 0.46212 | 0.35348 | 0.15378 |
| Llama LLMs | Llama-3.2-11B | 0.12117 | 0.10692 | 0.16869 | 0.16961 | 0.08713 | 0.21990 | 0.19622 | 0.74248 |
|  | Llama-3.2-11B-int4 | 0.06045 | 0.08514 | 0.12487 | 0.10797 | 0.20900 | 0.07194 | 0.10457 | 0.44601 |
| Gemma LLM | Gemma2-9B | 0.05169 | 0.06241 | 0.06571 | 0.11984 | 0.09811 | 0.10631 | 0.19532 | 0.05873 |
| Medical Models | huatuoGPT-o1-7B | 0.59108 | 0.73667 | 0.76914 | 0.67212 | 0.63584 | 0.80164 | 0.79377 | 0.82253 |
|  | huatuoGPT2-7B (Twice) | 0.94535 | 0.93690 | 0.86893 | 0.93889 | 0.89357 | 0.76743 | 0.88450 | 0.66301 |
|  | MMedLM2-7B (Twice) | 0.98573 | 0.96412 | 0.94738 | 0.79955 | **0.98993** | 0.98938 | 0.90811 | 0.87050 |
| DeepSeek LLMs | DeepSeek-32B | 0.55808 | 0.58118 | 0.64863 | 0.56164 | 0.53419 | 0.56164 | 0.58448 | 0.56390 |
|  | DeepSeek-R1 | 0.51069 | 0.56600 | 0.51637 | 0.55742 | 0.57854 | 0.71053 | 0.54475 | 0.56059 |
| **Task 3: Metabolic Syndrome Diagnosis (F1 Score)** | | | | | | | | | |
| Series | Models | Chinese Prompt | | | | English Prompt | | | |
|  |  | Prompt 1 | Prompt 2 | Prompt 3 | Prompt 4 | Prompt 1 | Prompt 2 | Prompt 3 | Prompt 4 |
| Qwen LLMs | Qwen2-2B | 0.49857 | 0.49788 | 0.49847 | 0.49788 | 0.48030 | 0.50107 | 0.49920 | 0.49788 |
|  | Qwen2.5-14B-int4 | 0.59815 | 0.60897 | 0.55137 | 0.50881 | 0.58483 | 0.59600 | 0.57327 | 0.52813 |
| Llama LLMs | Llama-3.2-11B | 0.50899 | 0.50412 | 0.51591 | 0.52587 | 0.51514 | 0.52293 | 0.52396 | 0.47119 |
|  | Llama-3.2-11B-int4 | 0.50354 | 0.49817 | 0.51587 | 0.51697 | 0.51479 | 0.50616 | 0.51452 | 0.52846 |
| Gemma LLM | Gemma2-9B | 0.50843 | 0.50904 | 0.50971 | 0.52076 | 0.51764 | 0.51781 | 0.51461 | 0.50994 |
| Medical Models | huatuoGPT-o1-7B | 0.61810 | 0.59890 | 0.63151 | 0.63783 | 0.61701 | 0.58733 | 0.62161 | 0.59188 |
|  | huatuoGPT2-7B (Twice) | 0.11789 | 0.11581 | 0.25835 | 0.15496 | 0.21504 | 0.34529 | 0.21455 | 0.37519 |
|  | MMedLM2-7B (Twice) | 0.04261 | 0.06985 | 0.13212 | 0.27535 | 0.02687 | 0.03348 | 0.16877 | 0.19124 |
| DeepSeek LLMs | DeepSeek-32B | 0.63495 | 0.62797 | **0.65004** | 0.62734 | 0.61730 | 0.63040 | 0.62365 | 0.62537 |
|  | DeepSeek-R1 | 0.61788 | 0.63228 | 0.61455 | 0.62938 | 0.60494 | 0.53817 | 0.59924 | 0.62552 |

**Notes:** The performance comparison metrics (including Accuracy, Recall or sensitivity, Specificity and F1-score) of the algorithm models on Task 3. Each row represents a model, and each column represents an evaluation metric. Values in **bold** denote the best-performing results.

**Table S6. The 95%CI of F1 scores of large language models on metabolic syndrome diagnosis task (Task 3).**

| **Task 3: Metabolic Syndrome Diagnosis (F1 Score--95%CI)** | | | | | | | | | |
| --- | --- | --- | --- | --- | --- | --- | --- | --- | --- |
| Series | Models | Chinese Prompt | | | | English Prompt | | | |
|  |  | Prompt 1 | Prompt 2 | Prompt 3 | Prompt 4 | Prompt 1 | Prompt 2 | Prompt 3 | Prompt 4 |
| Qwen LLMs | Qwen2-2B | [0.489, 0.509] | [0.488, 0.508] | [0.489, 0.508] | [0.488, 0.508] | [0.468, 0.492] | [0.491, 0.512] | [0.489, 0.509] | [0.489, 0.508] |
|  | Qwen2.5-14B-int4 | [0.588, 0.609] | [0.598, 0.620] | [0.541, 0.561] | [0.499, 0.520] | [0.574, 0.595] | [0.585, 0.606] | [0.563, 0.583] | [0.517, 0.537] |
| Llama LLMs | Llama-3.2-11B | [0.498, 0.519] | [0.494, 0.514] | [0.506, 0.526] | [0.516, 0.536] | [0.505, 0.525] | [0.513, 0.533] | [0.514, 0.534] | [0.456, 0.485] |
|  | Llama-3.2-11B-int4 | [0.494, 0.513] | [0.488, 0.508] | [0.505, 0.526] | [0.507, 0.527] | [0.504, 0.525] | [0.496, 0.515] | [0.505, 0.525] | [0.517, 0.539] |
| Gemma LLM | Gemma2-9B | [0.498, 0.518] | [0.499, 0.519] | [0.500, 0.519] | [0.511, 0.531] | [0.508, 0.528] | [0.508, 0.528] | [0.505, 0.524] | [0.500, 0.520] |
| Medical Models | huatuoGPT-o1-7B | [0.607, 0.629] | [0.586, 0.612] | [0.619, 0.645] | [0.627, 0.649] | [0.606, 0.628] | [0.573, 0.600] | [0.609, 0.634] | [0.579, 0.606] |
|  | huatuoGPT2-7B (Twice) | [0.105, 0.131] | [0.104, 0.128] | [0.244, 0.272] | [0.140, 0.170] | [0.200, 0.230] | [0.330, 0.360] | [0.199, 0.229] | [0.361, 0.389] |
|  | MMedLM2-7B (Twice) | [0.034, 0.052] | [0.059, 0.081] | [0.119, 0.146] | [0.260, 0.290] | [0.020, 0.035] | [0.026, 0.041] | [0.154, 0.183] | [0.177, 0.207] |
| DeepSeek LLMs | DeepSeek-32B | [0.624, 0.645] | [0.617, 0.638] | **[0.638, 0.660]** | [0.616, 0.638] | [0.606, 0.628] | [0.620, 0.640] | [0.612, 0.635] | [0.614, 0.636] |
|  | DeepSeek-R1 | [0.608, 0.628] | [0.621, 0.643] | [0.603, 0.626] | [0.618, 0.641] | [0.594, 0.616] | [0.524, 0.552] | [0.588, 0.610] | [0.615, 0.636] |

**Notes:** All values represented the 95% confidence intervals (CIs) for F1 scores. DeepSeek-32B, when paired with Chinese prompt3 (bold noted), exhibited the narrowest interval and the highest upper bound.
