# Supplementary material for: Real-world performance of open-source large language models in diabetes diagnosis
Source: Front Endocrinol (Lausanne). 2026 Mar 25;17:1747468. doi: 10.3389/fendo.2026.1747468 (PMC13056687; doi:10.3389/fendo.2026.1747468)
Supplement: Supplementary file 3 [file DataSheet3.docx]

**Task1-Diabetes Classification**

**English Prompts**

prompt_dia = {

    1: "What type of diabetes should this patient be diagnosed with?",

    2: "According to the 2025 ADA diabetes classification recommendations, what type of diabetes does the patient have?",

    3: """According to the 2025 ADA diabetes classification, different types of diabetes have the following characteristics:

    - **Type 1 Diabetes**:

      1) Onset at a young age (<35 years)

      2) Lean body type (BMI <25 kg/m²)

      3) Ketoacidosis at onset

      4) Poor pancreatic function (C-peptide <200 pmol/L)

      5) Regardless of the above features, if pancreatic autoantibodies are positive, a diagnosis of Type 1 diabetes can be made.

    - **Special Types of Diabetes**:

      1) **Monogenic Diabetes**:

         - **Neonatal Diabetes**: Diagnosed within the first 6 months after birth, requiring genetic testing.

         - **Maturity-Onset Diabetes of the Young (MODY)**:

           - Onset before 20 years old + negative pancreatic autoantibodies

           - Or onset between 20–30 years old + negative pancreatic autoantibodies + non-obese; persistently mildly elevated fasting blood glucose and HbA1c + first-degree family history

           - History of neonatal hyperinsulinemic hypoglycemia

           - Maternal inheritance, with associated hearing loss, optic atrophy, or skeletal muscle symptoms

           - Severe insulin resistance with prominent acanthosis nigricans disproportionate to obesity level

           - Congenital heart disease, gastrointestinal malformations, brain abnormalities, vision/hearing impairment, intellectual disabilities, growth retardation, severe diarrhea, kidney malformations, or other signs suggesting genetic mutations.

      2) **Pancreatic Diabetes (Type 3c Diabetes)**:

         - Secondary to pancreatic diseases (e.g., pancreatitis, pancreatic surgery, etc.)

         - Requires assessment of exocrine pancreatic insufficiency, pathological pancreatic imaging, and exclusion of autoimmune Type 1 diabetes.

      3) **Diabetes Associated with Other Conditions Leading to Hyperglycemia**

      4) **Drug-Induced Diabetes**: Due to medications that raise blood glucose levels.

    - **Gestational Diabetes**: Hyperglycemia occurring during pregnancy.

    - **Type 2 Diabetes**: If the above types are excluded, the diagnosis is Type 2 diabetes.

    Based on the above characteristics, what type of diabetes should this patient be diagnosed with?""",

    4: """According to the 2025 ADA diabetes classification process, the diagnostic reasoning for diabetes classification is as follows:

    1) First, determine whether the patient has gestational diabetes by assessing if they are pregnant.

       If the patient is pregnant and has elevated blood glucose levels, they can be diagnosed with gestational diabetes.

    2) If gestational diabetes is excluded, assess whether the patient might have a special type of diabetes:

       - Consider whether the patient has underlying conditions, such as exocrine pancreatic diseases (e.g., pancreatitis, pancreatic surgery, cystic fibrosis-related diabetes).

       - If no such conditions are present, evaluate whether the patient has other diseases that may cause hyperglycemia (e.g., thyroid disease, adrenal disease, pituitary disease, infections, etc.).

       - Check whether the patient is taking medications that could raise blood glucose (e.g., glucocorticoids, streptozotocin, immune checkpoint inhibitors, α-interferon, etc.).

       - Look for special signs (e.g., deafness, polycystic kidneys, distinctive facial features) that might suggest a special type of diabetes.

       - If the patient has a strong family history, with at least one first-degree relative affected over two consecutive generations, monogenic diabetes should be considered.

    3) If the above conditions do not apply, assess whether the patient has Type 1 diabetes:

       - Determine if the patient has immunological markers, such as positive pancreatic autoantibodies (GADA, IA2A, ZnT8A, etc.).

         If autoantibodies are positive, a diagnosis of Type 1 diabetes can be made.

       - If autoantibodies are negative, consider age of onset:

         - If onset occurs before 20 years old, accompanied by ketoacidosis or a need for insulin therapy for more than 6 months, and C-peptide <200 pmol/L, Type 1 diabetes is likely.

         - If onset occurs between 20–35 years old, the patient is non-obese, has mildly elevated fasting blood glucose and HbA1c, and has a first-degree family history, monogenic diabetes should be considered.

         - Genetic testing can help confirm monogenic diabetes in uncertain cases.

    4) **If none of the above criteria are met, Type 2 diabetes should be considered.**

    Based on the above diagnostic reasoning, what type of diabetes should this patient be diagnosed with?"""

}

additional_prompt = "You must choose exactly one option from the following: Type 1 diabetes, Type 2 diabetes, Specific Types of Diabetes, or Gestational Diabetes Mellitus. Directly output your choice without anything else. No additional explanation is needed."

**input_text form**

            input_text = f"Admission condition: {admission_condition}\n" \

                         f"Hospitalization process: {hospitalization_process}\n" \

                         f"Understand the content of the patient's admission condition and hospitalization process above in English.If you are a professional doctor," \

                         f"{question}\n"\

                         f"{additional_prompt}"

**Chinese prompts**

prompt_dia = {

    1: "该患者应诊断为哪种糖尿病类型？",

    2: "根据2025年版ADA糖尿病分型建议，患者的糖尿病属于哪一类型？",

    3: """根据2025年版ADA糖尿病分型，各型糖尿病患者具有以下特征：

    - **1型糖尿病**：

      1) 年轻起病（<35岁）

      2) 体型消瘦（BMI<25kg/m²）

      3) 酮症起病

      4) 胰岛功能较差（C肽<200 pmol/L）

      5) 无论是否具备以上特征，胰岛自身抗体为阳性时，即可诊断为1型糖尿病。

    - **特殊类型糖尿病**：

      1) **单基因糖尿病 (Monogenic Diabetes)**：

          - **新生儿糖尿病 (Neonatal Diabetes)**：出生后6个月内诊断，需基因检测。

          - **青年起病成人型糖尿病 (MODY)**：

            - 起病<20 岁+胰岛自身抗体阴性

            - 或起病 20~30 岁+胰岛自身抗体阴性+非肥胖;持续性轻度升高的空腹血糖和HbA1c + 一代家族史

            - 新生儿期有高胰岛素性低血糖症

            - 母系遗传，伴听力受损、视神经萎缩或骨骼肌表现

            - 与肥胖程度不符合的显著黑棘皮表现，存在严重胰岛素抵抗

            - 合并先天性心脏病、胃肠道缺陷、脑畸形、视力听力异常、智力发育迟缓、生长发育障碍、严重腹泻、肾发育异常或其他疑似基因突变相关的表现

      2) **胰腺外分泌疾病相关糖尿病 (Pancreatic Diabetes/Type 3c)**：

         - 继发于胰腺疾病（如胰腺炎、胰腺切除术等）

         - 需评估胰腺外分泌功能不全，病理胰腺影像学，并排除1型糖尿病的自身免疫因素

      3) **合并其他引起血糖升高的疾病**

      4) **服用引起血糖升高的药物**

    - **妊娠期高血糖**：妊娠期合并血糖升高

    - **2型糖尿病**：排除以上类型可诊断为2型糖尿病。

    根据上述特征，该患者应诊断为糖尿病的哪一类型？""",

    4: """根据2025年版ADA糖尿病分型流程，糖尿病分型诊断的思维链如下：

    1) 首先判断患者是否为妊娠期高血糖，评估患者是否为妊娠状态，

        如果患者为妊娠状态时且伴有血糖升高，患者即可诊断为妊娠期高血糖。

    2) 如果患者不符合妊娠期高血糖的诊断，则需要评估患者是否可能为特殊类型糖尿病：

       - 需要考虑患者是否有基础疾病，包括胰腺外分泌相关的疾病，如胰腺炎，胰腺手术，囊性纤维化相关胰腺疾病

       - 如果患者无以上基础疾病，需要考虑患者是否有其他引起血糖升高的疾病（如甲状腺疾病、肾上腺疾病、垂体疾病、感染等）？

       - 或者患者是否正在服用可能引起血糖升高的药物（如糖皮质激素、链脲菌素、免疫检查点抑制剂、α‑干扰素等）？

       - 另外需要评估患者是否有特殊体征（如耳聋、多囊肾、特殊面容），提示可能为特殊类型糖尿病；

       - 如果患者有明显的家族史特征，包括三代中连续两代至少有一位直系亲属为糖尿病。提示可能为单基因糖尿病。

    3) 如果患者不符合以上判断，需要评估患者是否为1型糖尿病：这需要根据起病年龄，体型，胰岛功能，血糖控制，自身免疫特征综合评估。

       - 首先判断患者是否有免疫学特征，即胰岛自身抗体阳性，包括GADA、IA2A、ZnT8A等，若抗体阳性可直接诊断为1型糖尿病。

       - 随后根据起病年龄判断（如果没有免疫学特征，或者胰岛自身抗体为阴性的患者）：

         - 若起病<20岁，伴有酮症或酮症酸中毒，起病后依赖胰岛素治疗>6个月，C肽 <200 pmol/L，需考虑为1型糖尿病。

         - 若起病年龄 20~35 岁、非肥胖，且表现为持续性轻度升高的空腹血糖和HbA1c，且具有一代家族史，需考虑单基因糖尿病（即特殊类型糖尿病）。

         - 对于可疑单基因糖尿病患者，可增加基因检测以明确诊断。

    4) **若不符合以上类型，则考虑为2型糖尿病**。

    按照上述诊断思维链的诊断流程，该患者应诊断为哪种糖尿病的哪一类型？"""

}

additional_prompt = "直接给出一个你诊断出的糖尿病类型，输出时只能选择这四种病型的其中一种：1型糖尿病，2型糖尿病，特殊类型糖尿病，妊娠期高血糖，且不要添加其他文字。"

**input_text form:**

            input_text = f"入院时情况：{admission_condition}\n" \

                         f"住院经过：{hospitalization_process}\n" \

                         f"以上是患者的入院时情况和住院经过，如果你是一位专业医生，" \

                         f"{question}\n"\

                         f"{additional_prompt}"

**Task2-diabetic kidney disease diagnosis**

**Chinese prompts**

prompt_DN = {

    1: "患者是否诊断糖尿病肾病？",

    2: "结合患者既往病史，根据尿白蛋白，肾小球滤过率情况，患者是否诊断糖尿病肾病？",

    3: "排除患者是否有其他引起尿白蛋白升高的病因，根据尿白蛋白/尿肌酐≥ 30mg/g 或24小时尿白蛋白排泄率AER ≥ 20 μ g/min，患者是否诊断糖尿病肾病？",

    4: """诊断糖尿病肾病需要患者在有糖尿病的诊断基础上，排除其他可能引起尿白蛋白升高的疾病，

        包括高血压，多种原因所导致肾炎，多囊肾病，梗阻性肾病，药物性肾损伤，

        出现非一过性尿白蛋白/尿肌酐≥ 30mg/g 或24小时尿白蛋白排泄率AER ≥ 20 μ g/min，

        根据以上标准，患者能否诊断糖尿病肾病？"""

}

additional_prompt = "只输出你的诊断结果：如果能够诊断糖尿病肾病，直接输出1，否则输出0。直接输出1或0，不需要额外的文字说明。"

**English prompts**

prompt_DN = {

    1: "Has the patient been diagnosed with diabetic nephropathy?",

    2: "Based on the patient's medical history, urine albumin levels, and glomerular filtration rate, can the patient be diagnosed with diabetic nephropathy?",

    3: "After ruling out other causes of elevated urine albumin, and considering urine albumin/creatinine ratio ≥ 30 mg/g or 24-hour urine albumin excretion rate (AER) ≥ 20 μg/min, can the patient be diagnosed with diabetic nephropathy?",

    4: """The diagnosis of diabetic nephropathy requires a confirmed diagnosis of diabetes and the exclusion of other potential causes of elevated urine albumin,

        including hypertension, various types of nephritis, polycystic kidney disease, obstructive nephropathy, and drug-induced kidney injury.

        The presence of persistent urine albumin/creatinine ratio ≥ 30 mg/g or 24-hour urine albumin excretion rate (AER) ≥ 20 μg/min is also required.

        Based on these criteria, can the patient be diagnosed with diabetic nephropathy?"""

}

additional_prompt = "Output only your diagnosis result: If diabetic nephropathy can be diagnosed, output 1 directly; otherwise, output 0. Output only 1 or 0, without any additional text."

**Task3-Metabolic Syndrome diagnosis**

**Chinese prompts**

prompt_Metabolic = {

    1: "患者是否诊断代谢综合征？",

    2: "患者根据CDS 2017年标准能否诊断代谢综合征？",

    3: "根据患者体质指数、腰围、血压、血脂、血糖状态，能否诊断代谢综合征？",

    4: """具备以下至少3项即可诊断代谢综合征 ：

            （1）腹型肥胖（即中心型肥胖）：腰围男性≥90 cm，女性≥85 cm；

            （2）高血糖：空腹血糖≥6.1 mmol/L或口服葡萄糖耐量试验（OGTT）2 h血糖≥7.8 mmol/L和（或）已确诊为糖尿病并治疗者；

            （3）血压增高：血压≥130/85 mmHg（1 mmHg=0.133 kPa）和（或）已确诊为高血压并治疗者；

            （4）空腹甘油三酯（TG）≥1.70 mmol/L；

            （5）空腹HDL-C<1.04 mmol/L。

        根据以上标准，患者能否诊断代谢综合征？"""

}

additional_prompt = "只输出你的诊断结果：如果能够诊断代谢综合征，直接输出1，否则输出0。直接输出1或0，不需要额外的文字说明。"

**English prompts**

prompt_Metabolic = {

    1: "Has the patient been diagnosed with metabolic syndrome?",

    2: "Can the patient be diagnosed with metabolic syndrome based on the CDS 2017 criteria?",

    3: "Based on the patient's BMI, waist circumference, blood pressure, blood lipids, and blood glucose status, can metabolic syndrome be diagnosed?",

    4: """A diagnosis of metabolic syndrome requires at least three of the following criteria:

            (1) Abdominal obesity (central obesity): waist circumference ≥90 cm for men, ≥85 cm for women;

            (2) Hyperglycemia: fasting blood glucose ≥6.1 mmol/L or 2-hour blood glucose ≥7.8 mmol/L in an oral glucose tolerance test (OGTT), and/or a confirmed diagnosis of diabetes with ongoing treatment;

            (3) Elevated blood pressure: blood pressure ≥130/85 mmHg (1 mmHg = 0.133 kPa), and/or a confirmed diagnosis of hypertension with ongoing treatment;

            (4) Fasting triglycerides (TG) ≥1.70 mmol/L;

            (5) Fasting HDL-C <1.04 mmol/L.

        Based on the above criteria, can the patient be diagnosed with metabolic syndrome?"""

}

additional_prompt = "Output only your diagnostic result: If metabolic syndrome can be diagnosed, output 1; otherwise, output 0. Output only 1 or 0, without any additional explanation."
