# Supplementary material for: Real-world performance of open-source large language models in diabetes diagnosis
Source: Front Endocrinol (Lausanne). 2026 Mar 25;17:1747468. doi: 10.3389/fendo.2026.1747468 (PMC13056687; doi:10.3389/fendo.2026.1747468)
Supplement: Supplementary file 4 [file DataSheet4.docx]

**Supplemental figures**

**Title:** Real-World Performance of Open-Source Large Language Models in Diabetes Diagnosis

**Figure S1.** Pairwise comparison of eight prompting strategies for DeepSeek-R1 using McNemar's test for Task 1(p < 0.05).

**Figure S2.** McNemar's test for pairwise performance differences among six LLMs using the "zh-Predict 1" prompt for Task 1 (p < 0.05).

**Figure S3.** Performance comparison of the evaluated large language models on the balanced dataset for Task1.

**Figure S4.** F1 score heatmap of models versus prompts for Task 1.

**Figure S5.** Peak F1-score performance for each model with its optimal prompt for Task 1. The models were ranked by their highest achieved F1-score.

**Figure S6.** Performance comparison of different prompt strategies of all models for Task 1 with balanced dataset.

**Figure S7.** Comparison of average performance between Chinese and English prompts for all the models on Task 1 with balanced data.

**Figure S8.** Comparison of F1-score distributions achieved by various models for the diagnosis of diabetic nephropathy under 8 prompting strategies.

**Figure S9.** Comparison of F1-score distributions achieved by various models for the diagnosis of metabolic syndrome under 8 prompting strategies.

**Figure S10.** Performance scaling of Qwen and DeepSeek models on Chinese (zh) and English (eng) tasks.

**Figure S11.** Comparative performance of the Qwen and DeepSeek model series.

**Figure S12.** Medical vs. general-purpose model performance (~7B scale) on three tasks (a to c).

**Figure S13.** Comparison of full-precision and 4-bit integer quantized (INT4) versions of the Qwen2.5-14B and Llama-3.2-11B models on the three tasks.

**Figure S14.** Performance benchmark of distilled DeepSeek models versus original Qwen models.

**Figure S15.** The effect of prompt optimization on the Macro F1-score of different models for the diabetes classification diagnosis task (Task 1).

**Figure S16.** The effect of prompt optimization on the Macro F1-score of different models for the diabetic kidney disease diagnosis task (Task 2).

**Figure S17.** The effect of prompt optimization on the Macro F1-score of different models for the metabolic syndrome diagnosis task (Task 3).

**Table S18.** Comparison of missing rates for diagnostic clinical indicators in DKD (A) and MetS (B).


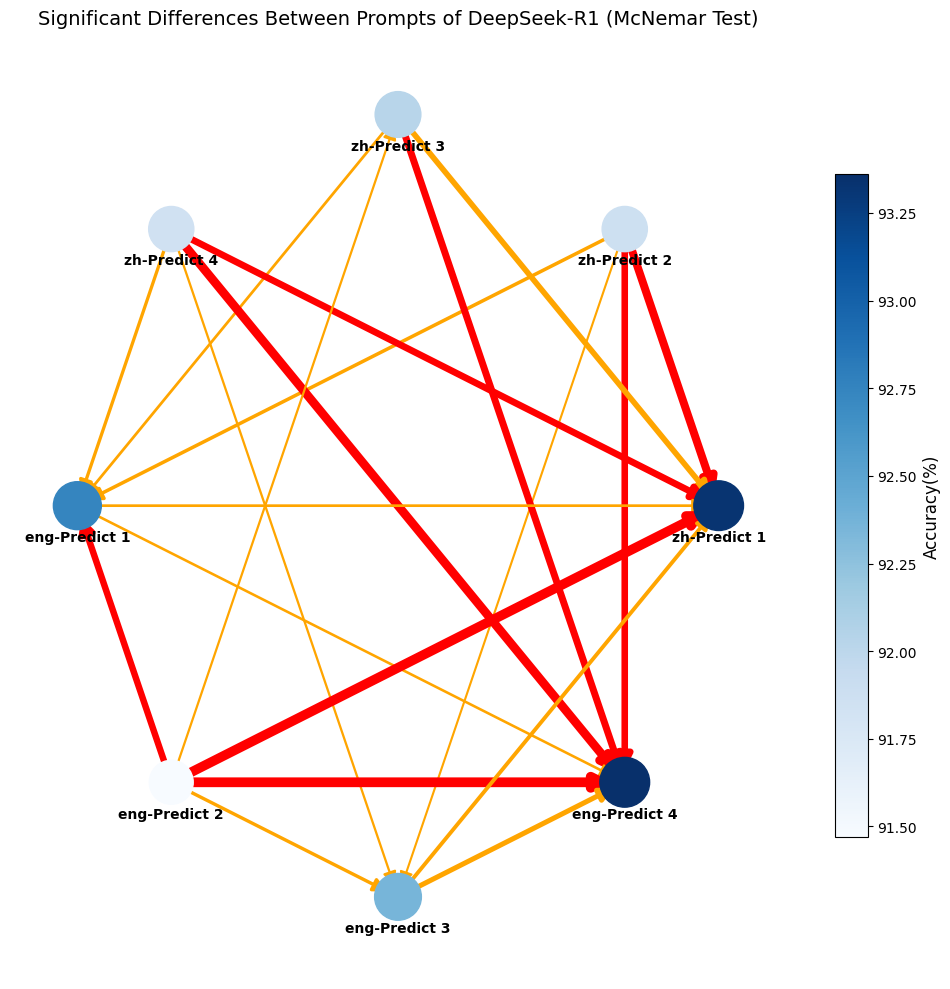


**Figure S1. Pairwise comparison of eight prompting strategies for DeepSeek-R1 using McNemar's test for Task 1(p < 0.05).** Key findings showed that: (1) The "zh-Predict 1" prompt yielded a significant accuracy improvement over most alternatives. (2) The "eng-Predict 4" prompt was the most effective among the English variants in significantly different pairs.


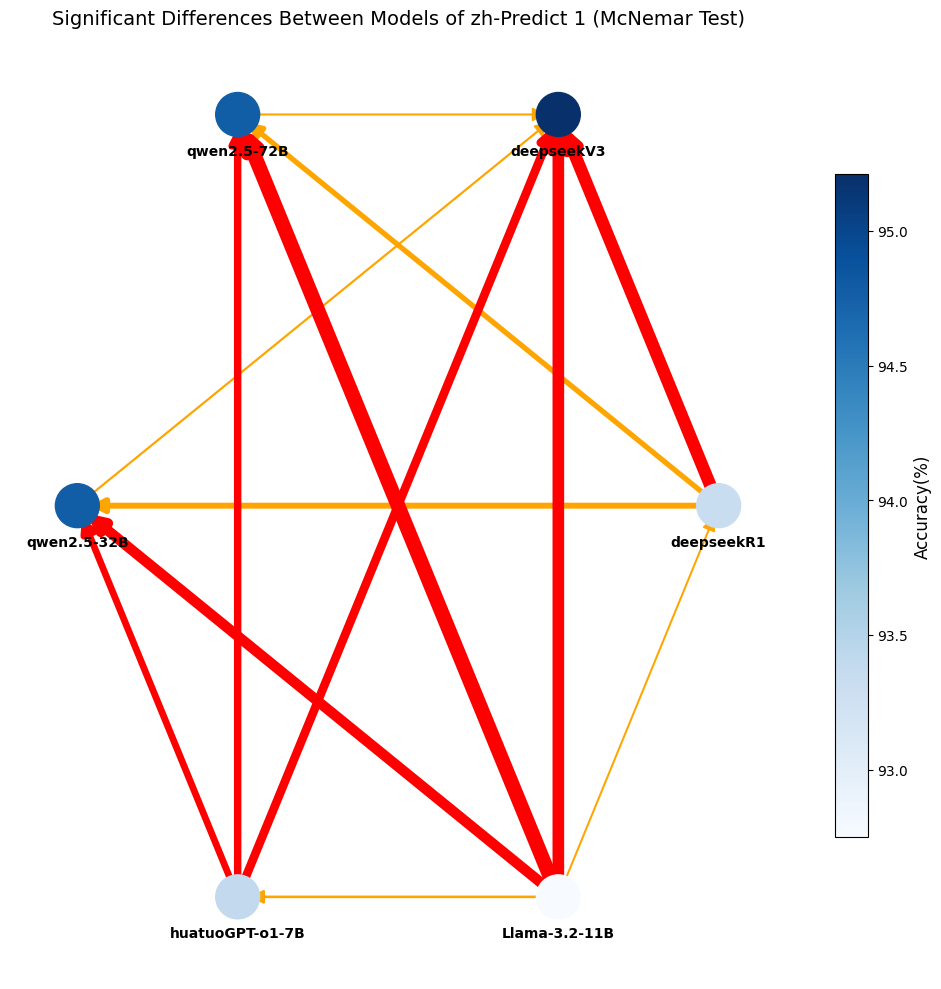


**Figure S2. McNemar's test for pairwise performance differences among six LLMs using the "zh-Predict 1" prompt for Task 1 (p < 0.05).** Directed edges denoted a statistically significant performance advantage. The graph illustrated the clear superiority of DeepSeek-V3. The Qwen2.5-72B and Qwen2.5-32B models also showed robust performance against several competitors. The models compared were from the DeepSeek, Qwen2.5, Llama-3.2, and huatuoGPT-o1 series.


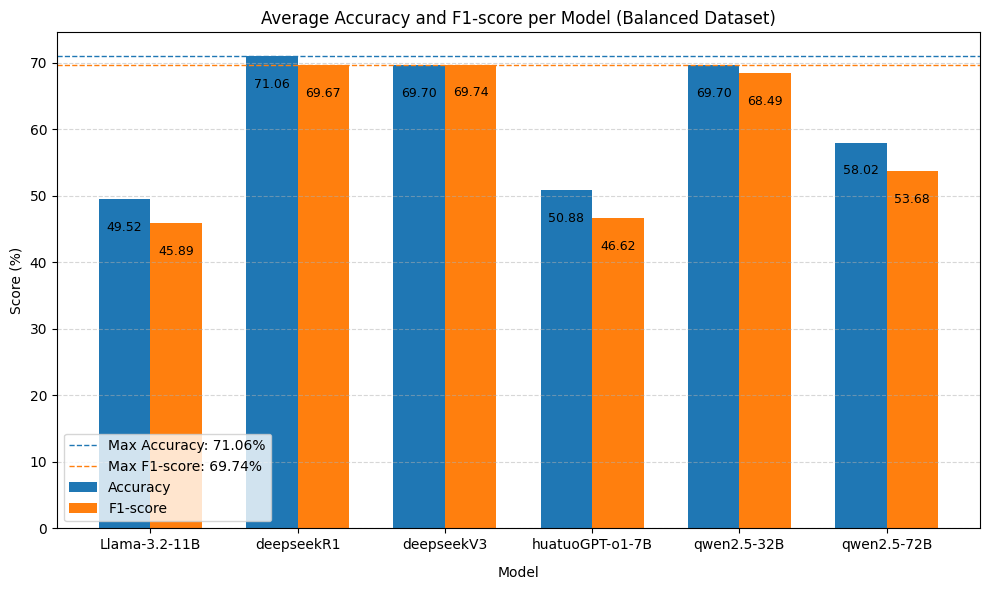


**Figure S3.** Performance comparison of the evaluated large language models on the balanced dataset for Task1. The chart displayed the average Accuracy (blue bars) and F1-score (orange bars).


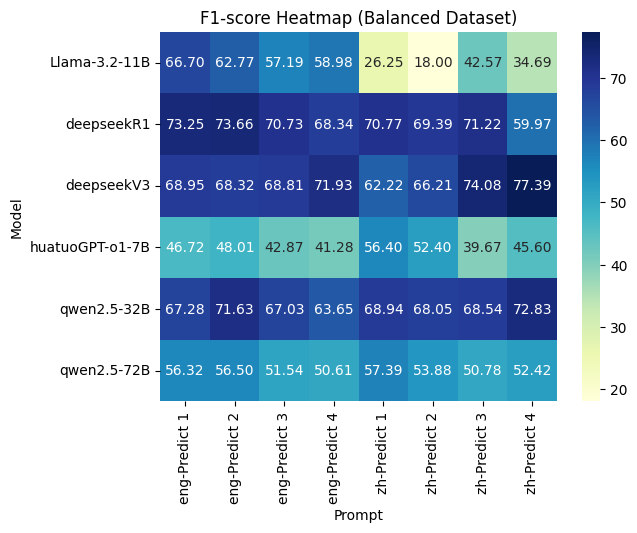


**Figure S4. F1 score heatmap of models versus prompts for Task 1.** The figure displays the accuracy and F1-score for each model-prompt pair for Task 1 in balanced data, visually representing model sensitivity to prompting strategies. Color intensity corresponds to performance.


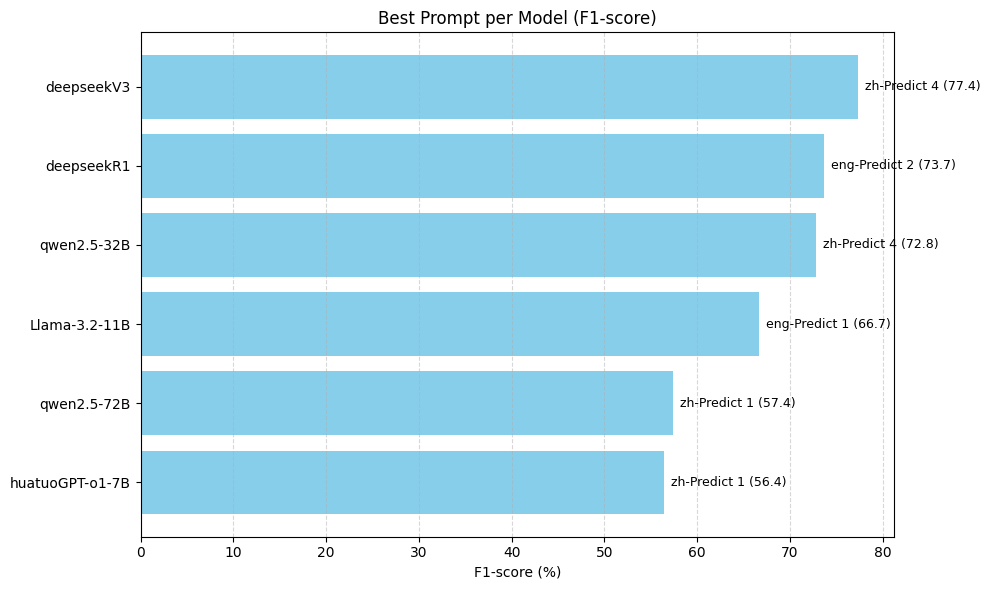


**Figure S5.** Peak F1-score performance for each model with its optimal prompt for Task 1. The models were ranked by their highest achieved F1-score.


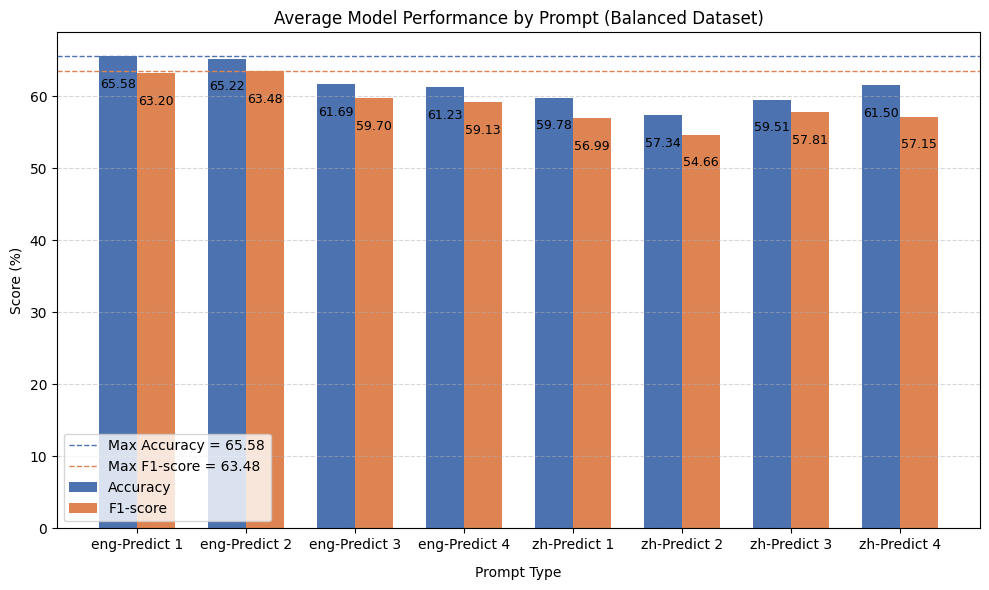


**Figure S6.** **Performance comparison of different prompt strategies of all models for Task 1 with balanced dataset.** The chart displayed the average accuracy (blue bars) and F1-score (orange bars) for each prompt, calculated across all evaluated models. English prompts, particularly English prompt 1 and 2, generally yielded higher performance than the Chinese prompts.


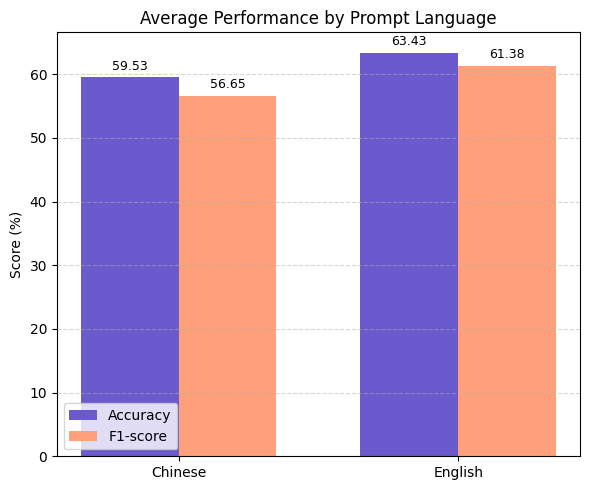


**Figure S7. Comparison of average performance between Chinese and English prompts for all the models on Task 1 with balanced data**. The metrics were calculated by averaging the performance of accuracy and F1-score across all models and prompts within each language category. English prompts demonstrated superior performance on both average Accuracy (63.43% vs. 59.53%) and F1-score (61.38% vs. 56.65%).


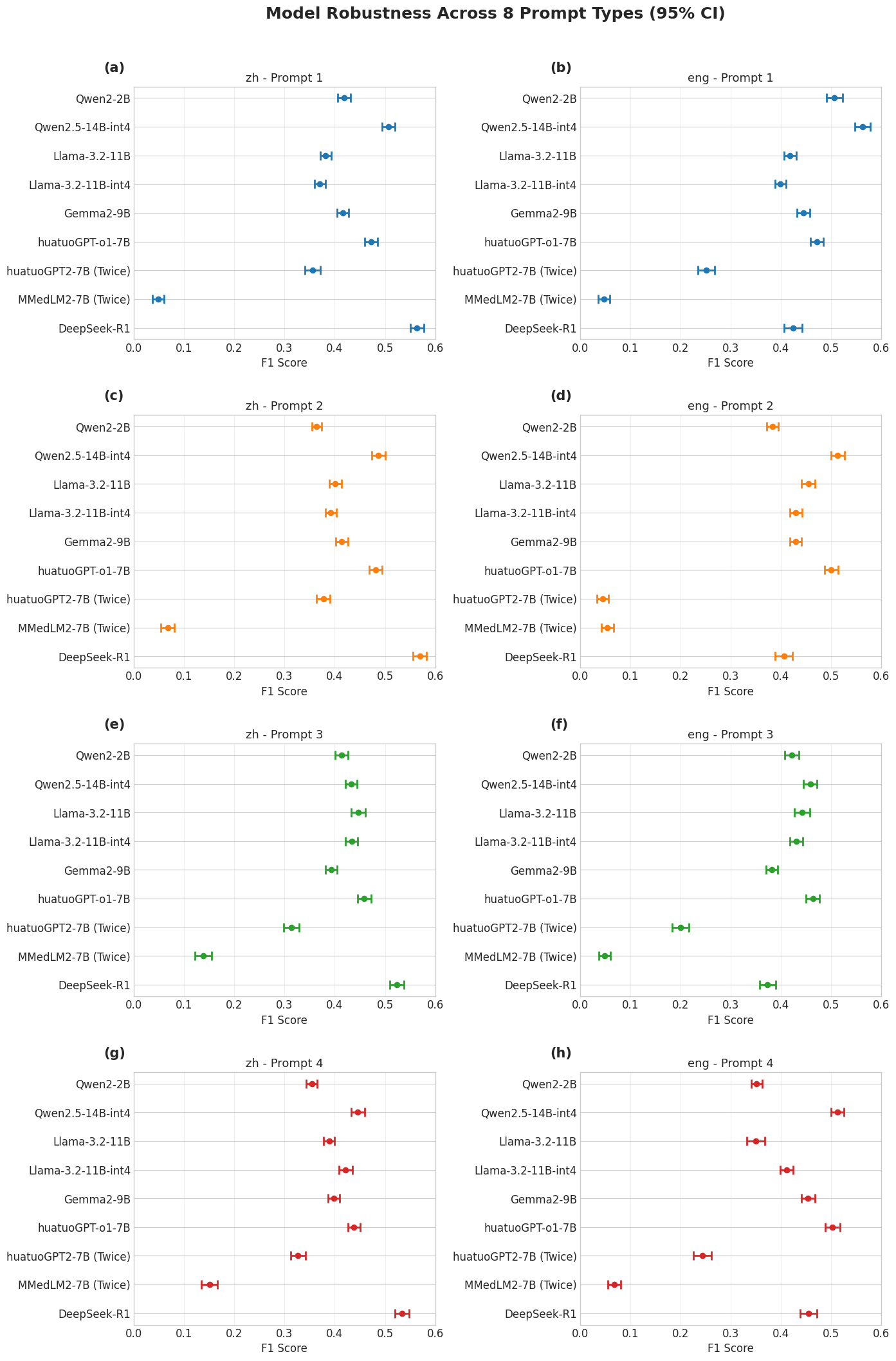


**Figure S8.** Comparison of F1-score distributions achieved by various models for the diagnosis of diabetic nephropathy under (a) Chinese prompt 1, (b) English prompt 1, (c) Chinese prompt 2, (d) English prompt 2, (e) Chinese prompt 3, (f) English prompt 3, (g) Chinese prompt 4, and (h) English prompt 4.


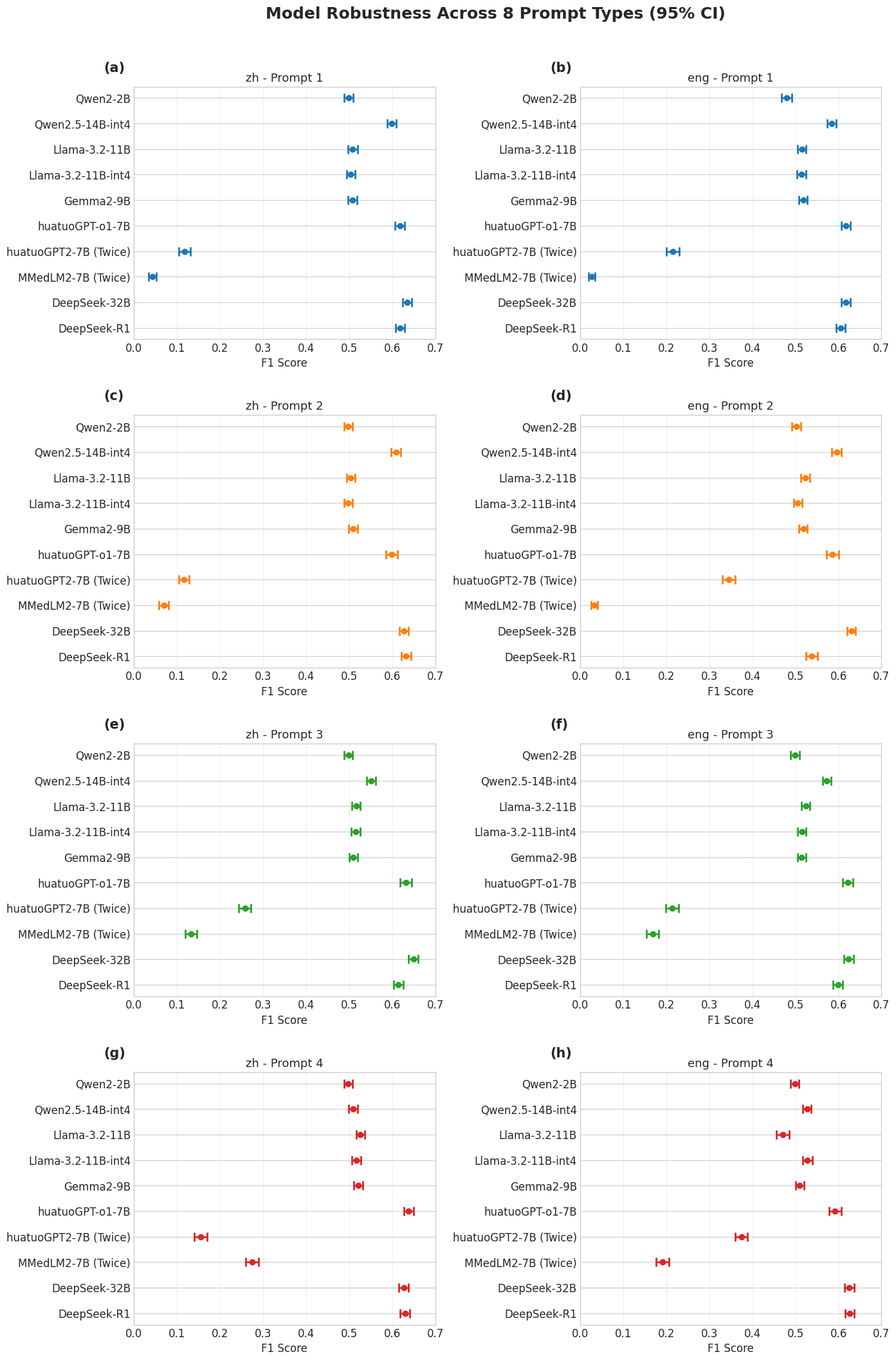


**Figure S9.** Comparison of F1-score distributions achieved by various models for the diagnosis of metabolic syndrome under (a) Chinese prompt 1, (b) English prompt 1, (c) Chinese prompt 2, (d) English prompt 2, (e) Chinese prompt 3, (f) English prompt 3, (g) Chinese prompt 4, and (h) English prompt 4.


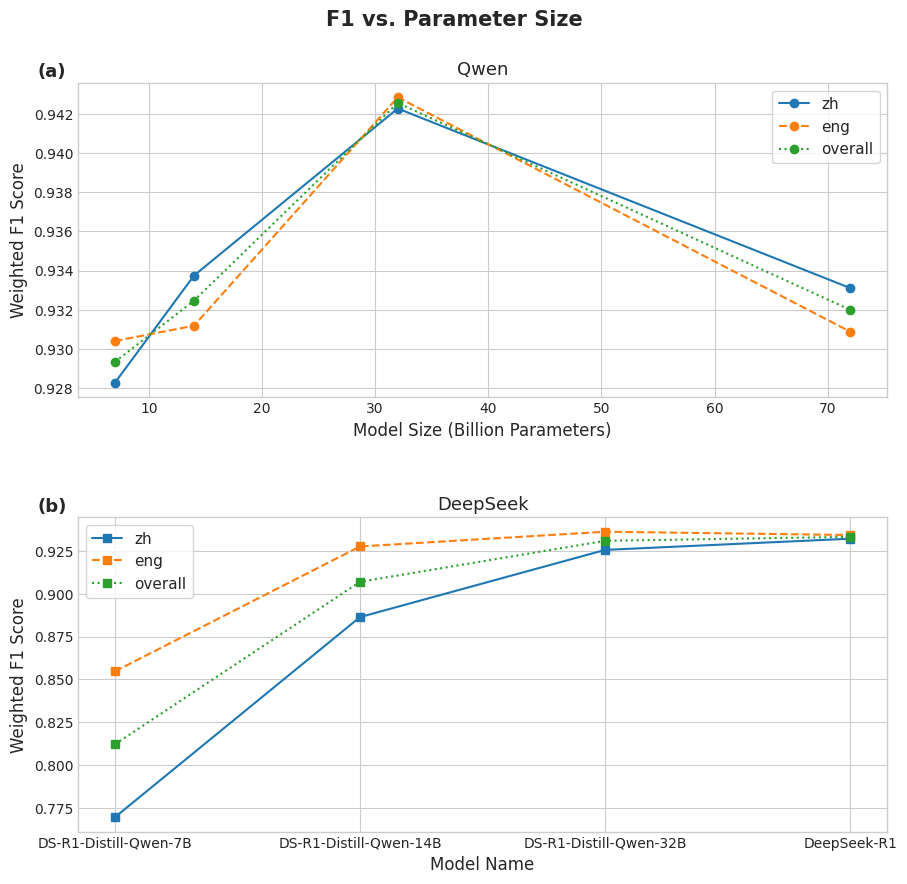


**Figure S10. Performance scaling of Qwen and DeepSeek models on Chinese (zh) and English (eng) tasks.** The plot showed the average Weighted F1 score as a function of model size. While a general positive trend was observed, the data revealed two key phenomena: a performance plateau in the Qwen series after the 32B model**(a)**, and diminishing returned for the DeepSeek series, where the largest model (671B) offered only marginal gains and was even slightly outperformed by a smaller 32B model in English **(b)**.


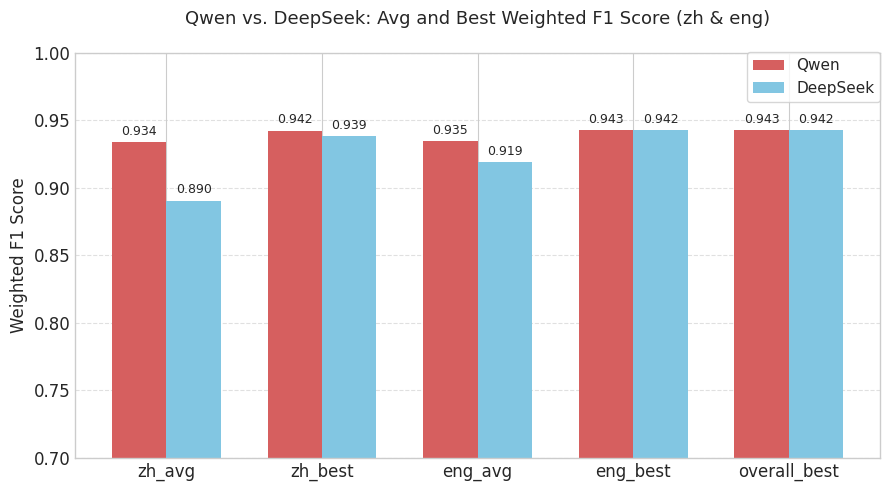


**Figure S11. Comparative performance of the Qwen and DeepSeek model series.** The bar chart displayed the mean and optimal (best) average Weighted F1 scores for each model family on Task 1. The comparison was shown separately for Chinese (zh) and English (eng) prompts. The results indicated that the Qwen series consistently outperformed the DeepSeek series in both average and optimal performance metrics across both languages.


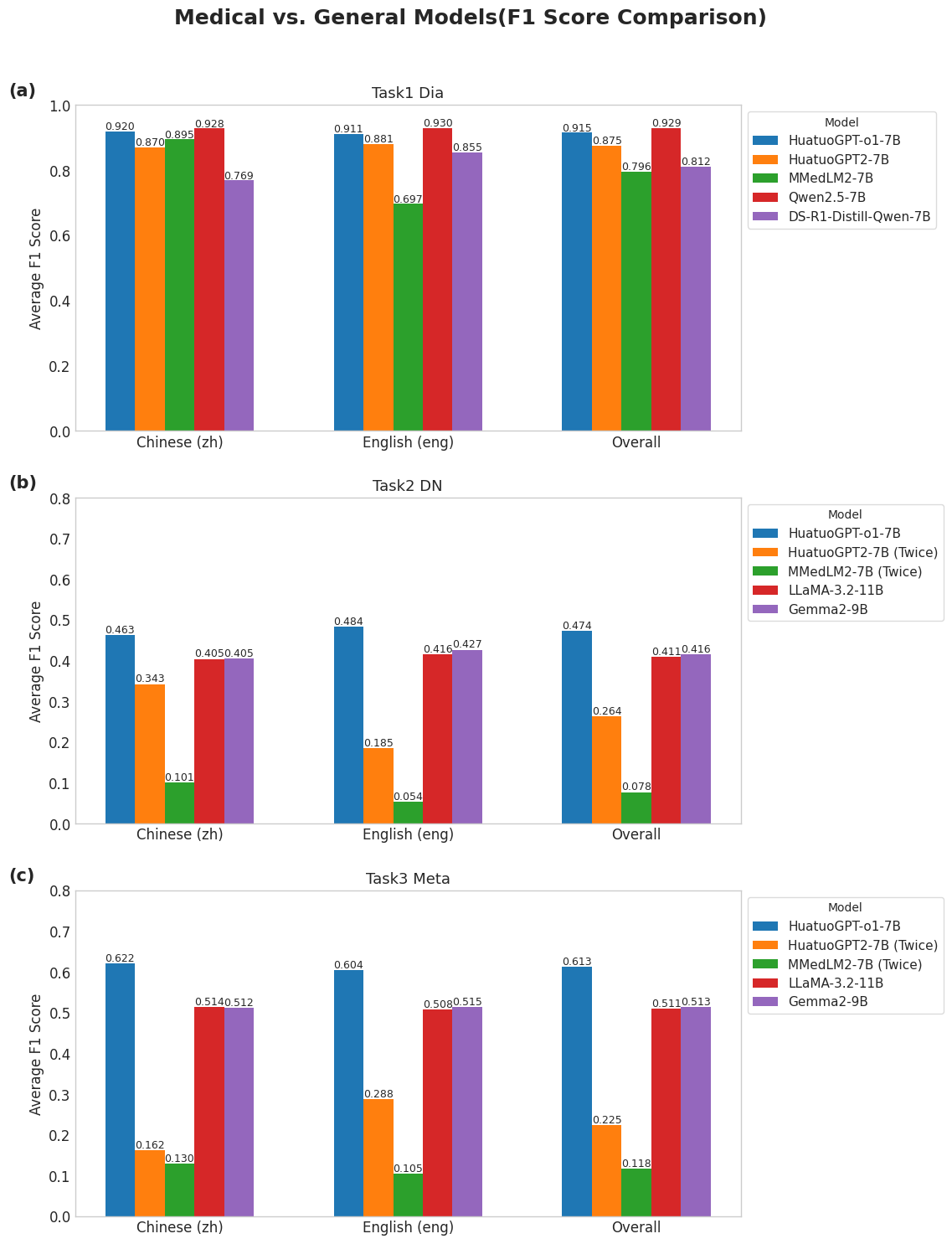


**Figure S12. Medical vs. general-purpose model performance (~7B scale) on three tasks (a to c).**The chart compared medically fine-tuned models (HuatuoGPT, MMedLM2) against general-purpose models. Performance was broken down by language (Chinese, English) and overall, allowing for a direct assessment of the benefits of domain-specific training.


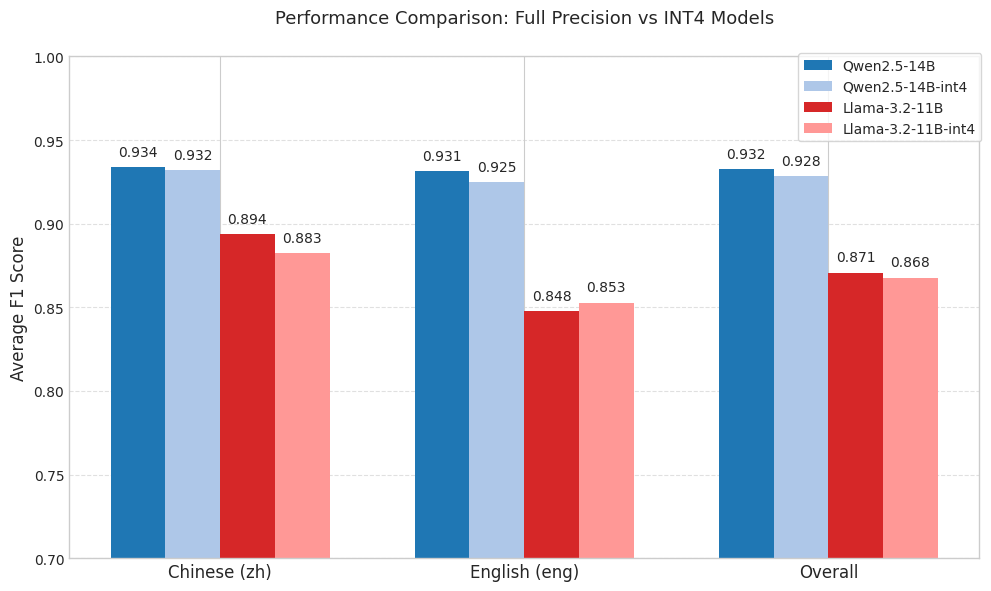


**Figure S13. Comparison of full-precision and 4-bit integer quantized (INT4) versions of the Qwen2.5-14B and Llama-3.2-11B models on the three tasks.** Performance was measured by the average weighted F1-score, evaluated across scenarios using Chinese, English, and aggregate overall prompts.


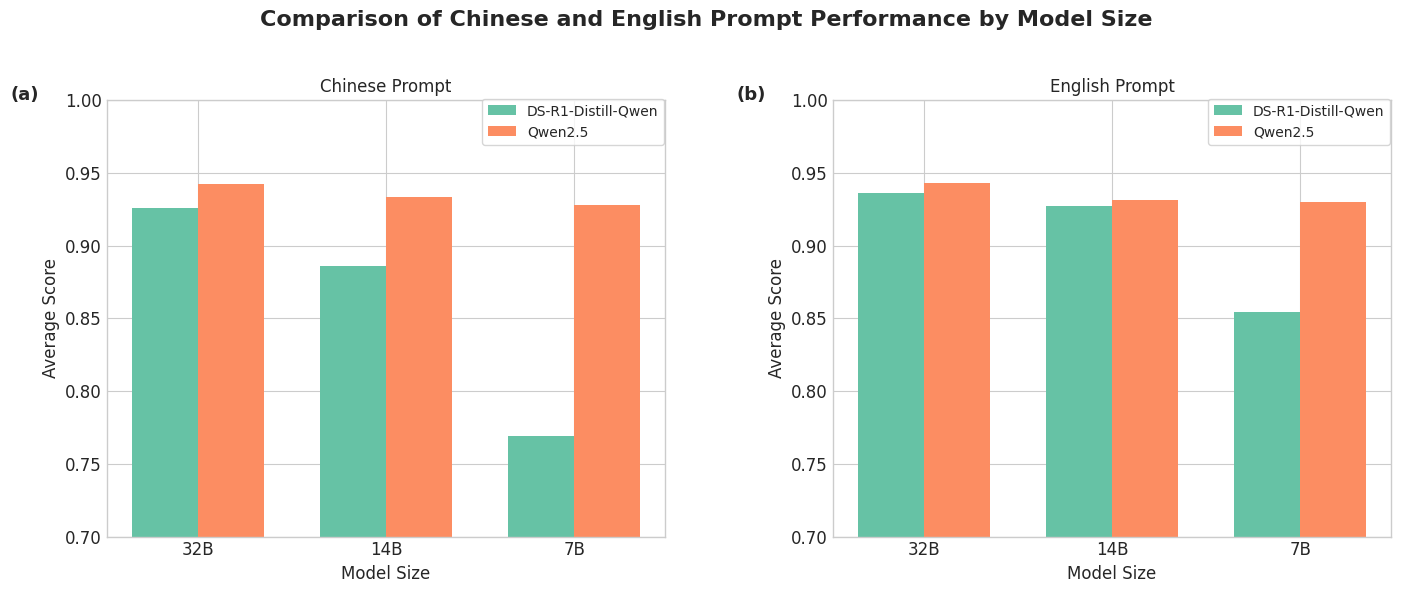


**Figure S14. Performance benchmark of distilled DeepSeek models versus original Qwen models.** The plots compared distilled DeepSeek series models (DS-R1-Distill-Qwen) with the original Qwen 2.5 models of corresponding parameter scales using **(a)**Chinese and **(b)**English prompts respectively.


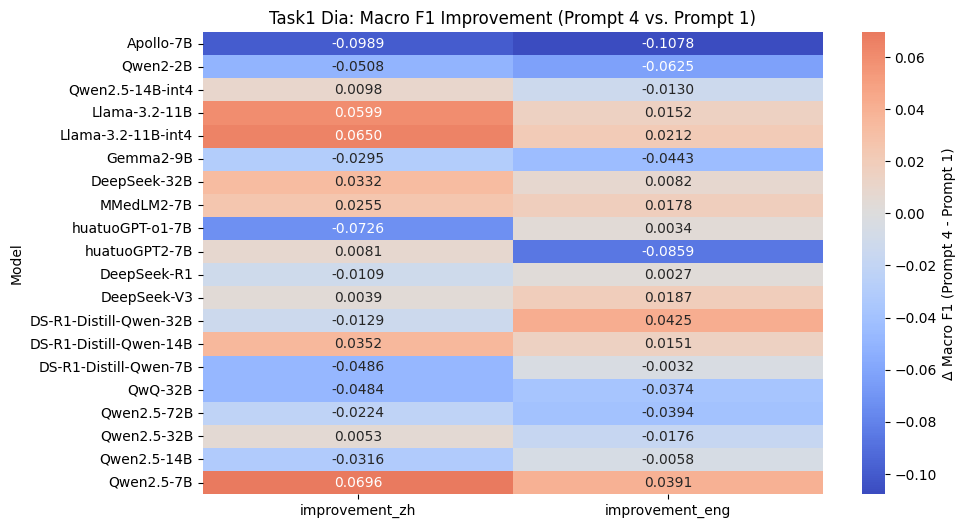


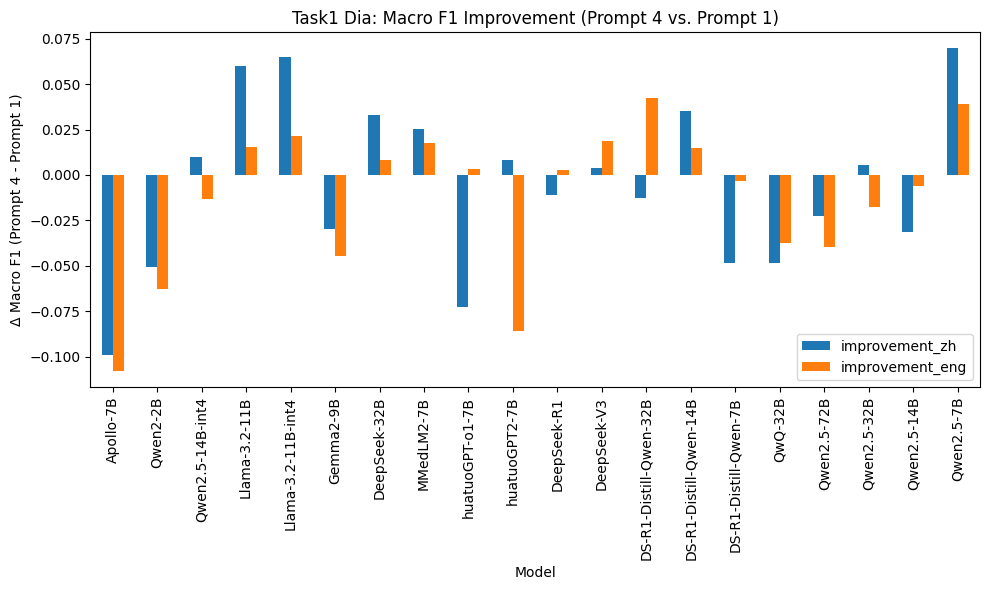


**Figure S15. The effect of prompt optimization on the Macro F1-score of different models for the diabetes classification diagnosis task (Task 1).** The figure plotted the performance delta achieved by using Prompt 4 relative to a baseline (Prompt 1). The performance of Qwen2.5-7B, Llama-3-2.11B, Llama-3-2.11B-int4 and DS-R1-Distill-Qwen-32B was improved under both Chinese and English conditions.


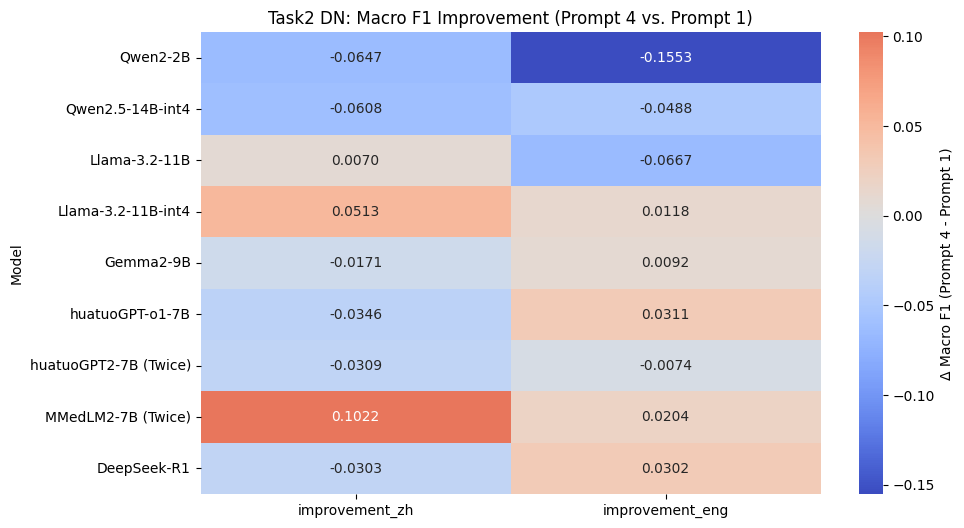


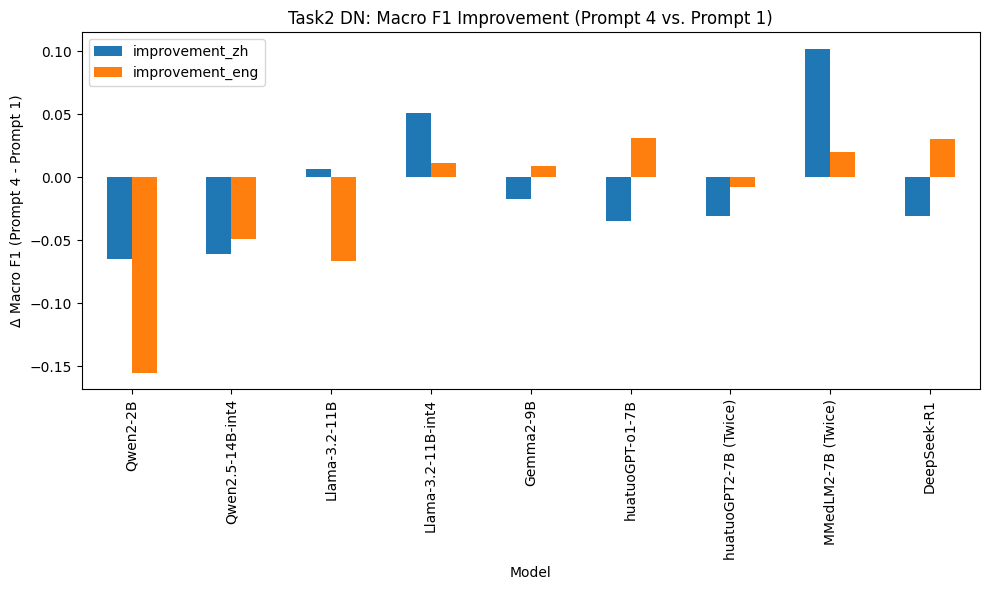


**Figure S16. The effect of prompt optimization on the Macro F1-score of different models for the** **diabetic kidney disease diagnosis task (Task 2).** The CoT strategy showed inconsistent effects, providing only slight gains in specific cases (Gemma2-English and Llama3.2-Chinese) while sometimes harming performance. However, its advantage over the baseline (Prompt 1) was more significant in the context of English-language tasks for a subset of models.


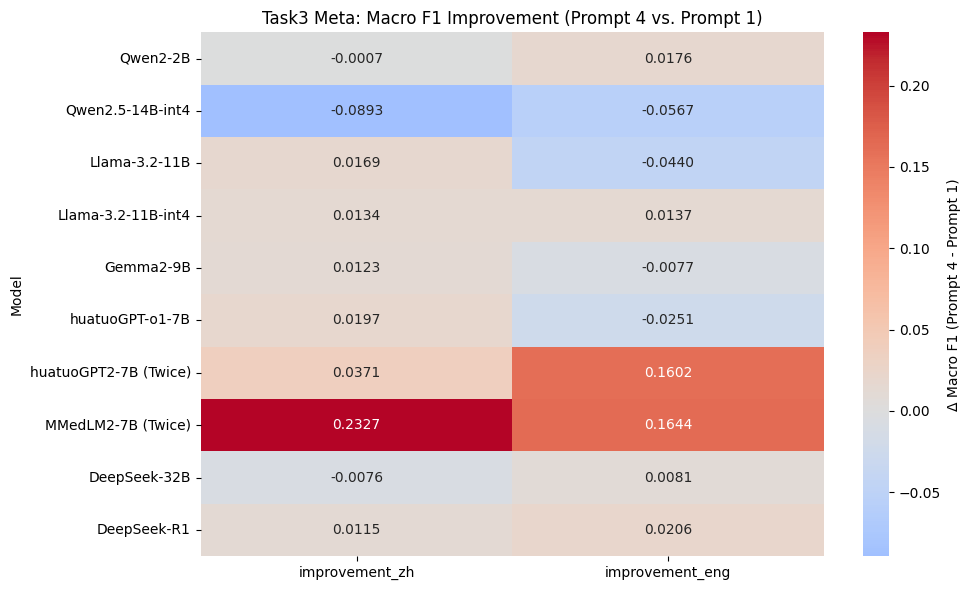


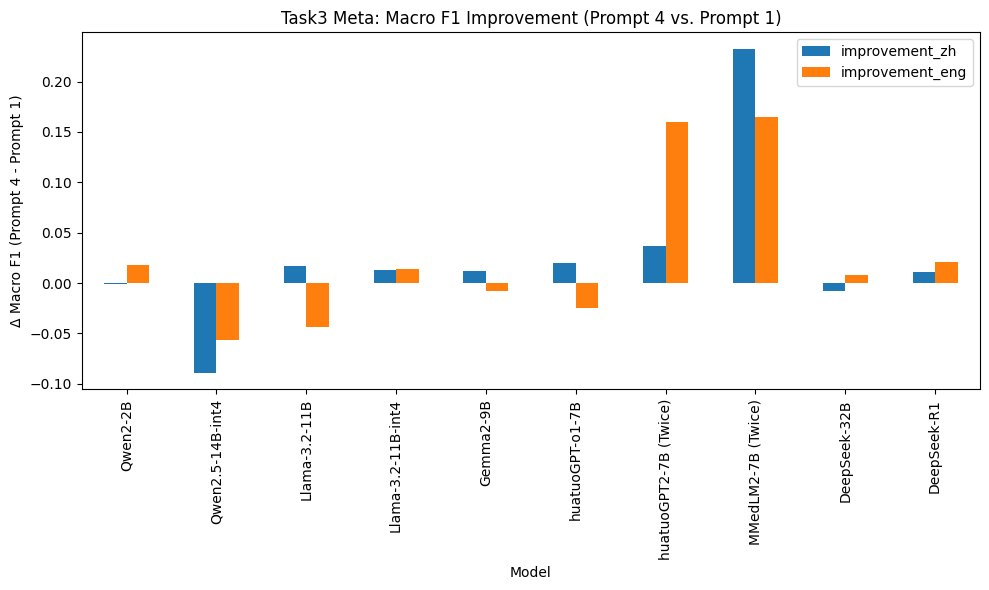


**Figure S17. The effect of prompt optimization on the Macro F1-score of different models for the metabolic syndrome diagnosis task (Task 3).** The figure plotted the performance delta achieved by using Prompt 4 relative to a baseline (Prompt 1) under both Chinese and English conditions. The CoT boosted performance for smaller or reasoning-sensitive models (MMedLM2-7B and huatuoGPT2-7B).


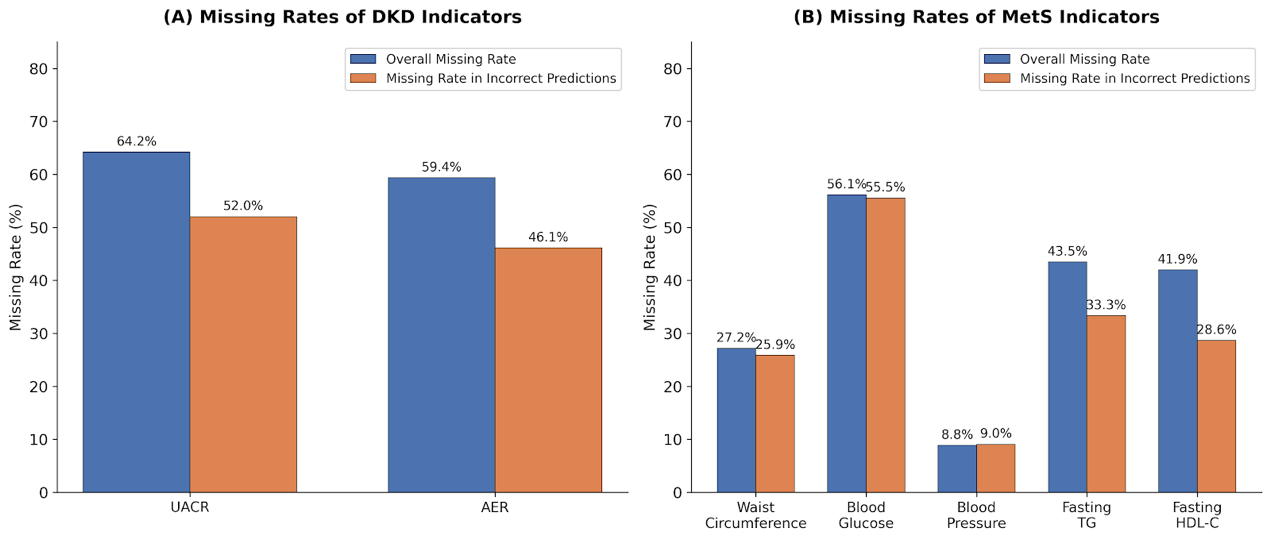


**Table S18. Comparison of missing rates for diagnostic clinical indicators in DKD (A) and MetS (B).** (A) represents the missing rates of key indicators (UACR and AER) for DN diagnosis, and (B) shows the missing rates of five core clinical indicators for MetS diagnosis. In both panels, the blue bars represent the overall missing rate in the entire dataset (n=11329), while the orange bars represent the missing rate exclusively within the subset of cases where the model yielded incorrect predictions (n=2482 for DKD, n=3725 for MetS).
